# C-C motif chemokine receptor 2 inhibition reduces liver fibrosis by restoring the immune cell landscape

**DOI:** 10.7150/ijbs.83530

**Published:** 2023-05-08

**Authors:** Yangkun Guo, Chong Zhao, Wenting Dai, Bowen Wang, Enjiang Lai, Yang Xiao, Chengwei Tang, Zhiyin Huang, Jinhang Gao

**Affiliations:** 1 Lab of Gastroenterology and Hepatology, State Key Laboratory of Biotherapy, West China Hospital, Sichuan University, Chengdu, China.; 2 Department of Gastroenterology; West China Hospital, Sichuan University, Chengdu, China.; 3 Department of Gastroenterology; General Hospital of Tibet Military Command, Lhasa, China.

**Keywords:** single-cell RNA sequencing, macrophage, neutrophil, STAT1, NFκB, ERK

## Abstract

The accumulation of extracellular matrix (ECM) proteins in the liver leads to liver fibrosis and end-stage liver cirrhosis. C-C motif chemokine receptor 2 (CCR2) is an attractive target for treating liver fibrosis. However, limited investigations have been conducted to explore the mechanism by which CCR2 inhibition reduces ECM accumulation and liver fibrosis, which is the focus of this study. Liver injury and liver fibrosis were induced by carbon tetrachloride (CCl_4_) in wild-type mice and *Ccr2* knockout (*Ccr2^-/-^*) mice. CCR2 was upregulated in murine and human fibrotic livers. Pharmacological CCR2 inhibition with cenicriviroc (CVC) reduced ECM accumulation and liver fibrosis in prevention and treatment administration. In single-cell RNA sequencing (scRNA-seq), CVC was demonstrated to alleviate liver fibrosis by restoring the macrophage and neutrophil landscape. CVC administration and CCR2 deletion can also inhibit the hepatic accumulation of inflammatory FSCN1^+^ macrophages and HERC6^+^ neutrophils. Pathway analysis indicated that the STAT1, NFκB, and ERK signaling pathways might be involved in the antifibrotic effects of CVC. Consistently, *Ccr2* knockout decreased phosphorylated STAT1, NFκB, and ERK in the liver.* In vitro*, CVC could transcriptionally suppress crucial profibrotic genes (*Xaf1*, *Slfn4*, *Slfn8, Ifi213*, and *Il1β*) in macrophages by inactivating the STAT1/NFκB/ERK signaling pathways. In conclusion, this study depicts a novel mechanism by which CVC alleviates ECM accumulation in liver fibrosis by restoring the immune cell landscape. CVC can inhibit profibrotic gene transcription *via* inactivating the CCR2-STAT1/NFκB/ERK signaling pathways.

## Introduction

In the context of chronic liver injuries, the accumulation of extracellular matrix (ECM) proteins in the liver leads to liver fibrosis and its end-stage liver cirrhosis.^1^ Liver cirrhosis is currently the 11^th^ leading cause of death worldwide due to its poor prognosis.^2^-^4^ Although many investigations have been conducted to clarify the mechanism and treatment of liver cirrhosis, no drugs with satisfactory anti-fibrosis effects have been approved.^5^ Immune cells have been demonstrated to be crucially involved in proinflammatory signaling pathways and ECM accumulation in liver fibrosis.^6^-^11^ In the injured liver, liver sinusoidal endothelial cells (LSECs) rapidly respond to injuries by recruiting other immune cells to the liver, eventually constructing a proinflammatory and profibrotic ECM deposition microenvironment.^8^, ^11^-^13^ Of these recruited immune cells, macrophages and neutrophils are the predominant cells that are distributed in the fibrotic septa and promote liver fibrosis by secreting proinflammatory mediators.^9^-^12^ Thus, therapies that target blocking immune cell infiltration have served as a new strategy for the clinical treatment of liver cirrhosis. However, limited therapeutic targets have been explored.

Monocyte-derived macrophages (MDMs), the major immune cell responses to liver injury, can influence ECM accumulation in liver fibrogenesis and its regression^14^-^17^. C-C motif chemokine receptor 2 (CCR2) is a classic G-protein-coupled transmembrane receptor distributed on immune cells, especially on MDMs.^18^ When binding to its ligand C-C motif chemokine ligand 2 (CCL2), CCR2 controls monocyte migration from the bone marrow into the liver and initiates the development of many liver diseases.^19^, ^20^ We have previously verified that CCR2^+^ monocytes extensively accumulate in the fibrotic liver, and suppressing CCR2^+^ monocyte infiltration by blocking CCL2 could effectively ameliorate ECM accumulation and liver fibrosis.^12^ Consistently, CCR2^+^ MDMs have been verified to participate in liver fibrosis and angiogenesis.^12^, ^21^-^23^ Thus, CCR2 is supposed to be an attractive target for the clinical treatment of liver cirrhosis.

Cenicriviroc (CVC) is an oral, dual antagonist of CCR2/CCR5 that was first developed to treat human immunodeficiency virus (HIV) infection.^24^, ^25^ Experimentally, CVC reduces hepatic steatosis and fibrosis by inhibiting the recruitment of Ly-6C^high^ monocytes and macrophages in murine nonalcoholic steatohepatitis (NASH) and alcohol-related liver disease (ALD).^15^, ^25^, ^26^ In a randomized, placebo-controlled trial and a phase IIb clinical trial, CVC has been successfully used to treat NASH and NASH-associated liver fibrosis.^27^, ^28^ However, CVC showed no significant improvement in liver fibrosis in a recent phase III clinical trial, which questioned the beneficial effects of CVC on human fibrosis.^29^ These controversial clinical and experimental data inspire us to explore the in-depth cellular and molecular mechanisms of CVC in liver fibrosis.

CVC was previously reported to attenuate liver fibrosis by inhibiting hepatic immune cell infiltration and angiogenesis.^23^, ^25^ Unlike previous reports in which CVC could attenuate liver fibrosis in NASH and ALD models, this study aimed to explore the antifibrotic role and mechanism of CVC in carbon tetrachloride (CCl_4_)-induced murine liver fibrosis. Indeed, both prevention and treatment administration of CVC can attenuate ECM accumulation in liver fibrosis. Single-cell RNA sequencing (scRNA-seq) and bulk RNA sequencing indicate that CVC reduces liver fibrosis by inhibiting hepatic accumulation of inflammatory fascin actin-bundling protein 1 (FSCN1)^+^ macrophages and HECT and RLD domain containing E3 ubiquitin protein ligase family member 6 (HERC6)^+^ neutrophils. CVC exerts antifibrotic and anti-inflammatory effects by transcriptionally inhibiting profibrotic genes (*Xaf1*, *Slfn4*, *Slfn8, Ifi213*, and *Il1β*) through inactivating the signal transducer and activator of transcription 1 (STAT1)/nuclear factor-κB (NFκB)/extracellular regulated protein kinase (ERK) signaling pathways.

## Materials and Methods

### Mice and induction of liver fibrosis

All animal experiments were approved by the Animal Care and Use Committee of West China Hospital, Sichuan University. Wild-type C57BL/6 mice were purchased from GemPharmatech (Nanjing, China). *Ccr2* knockout (*Ccr2^-/-^*) mice were a gift from Jing Wang (Shanghai Jiaotong University). Liver fibrosis was induced by CCl_4_ (1 μL/g body weight, Sigma‒Aldrich #319961) intraperitoneally (i.p.) injection. Mice in the control group (olive oil group) were i.p. injected with olive oil for 6 weeks. For the murine preventive model, wild-type mice were i.p. administered 4 dosages of CVC (15 mg/kg body weight, Selleck #S8512) and 2 dosages of CCl_4_ per week for 6 consecutive weeks. For the murine treatment model, mice were i.p. injected with CCl_4_ for 3 weeks to induce liver fibrosis, and then 4 dosages of CVC and 2 dosages of CCl_4_ per week were administered for another 3 weeks. For the acute liver injury experiment in *Ccr2^-/-^* mice, 2 doses of CCl_4_ were i.p. injected. All mice were sacrificed 48 hours by an overdose of pentobarbital sodium after the final injection, and livers were taken for subsequent experiments.

### Human liver tissues

Seven normal control and 11 cirrhotic livers were collected during hepatectomy at West China Hospital. All research was conducted following both the Declaration of Helsinki and Istanbul. The study was approved by the Ethical Committee of West China Hospital and registered in the Chinese Clinical Trial Registry (ChiCTR2200063108). Written informed consent forms were received from all patients. The detailed clinical information is listed in Supporting Table S1.

### Single-cell RNA sequencing (scRNA-seq) and bioinformatics analysis

scRNA-seq and bioinformatics analysis were performed by Shanghai OE Biotech Inc. (Shanghai, China). Three murine livers from 3 groups of the murine preventive model were mixed and assigned to scRNA. The raw scRNA-seq data have been deposited in the NCBI Gene Expression Omnibus (GSE218496). The detailed procedure is listed in the Supporting Materials.

### Statistical analysis

All data are presented as the mean ± standard deviation and were analyzed using GraphPad software (version 8, GraphPad Software Inc., La Jolla, CA, USA). One-way ANOVA for multiple-group comparisons followed by Bonferroni's *post hoc* test and *t*-test were used to analyze the data. A *p*-value <0.05 was considered statistically significant.

The Supporting Materials include scRNA-seq, RNA sequencing, bioinformatic analysis of the public GEO dataset (GSE136103 and GSE157088),^30^, ^31^ hematoxylin and eosin (H&E) staining, Sirius red staining, immunohistochemistry (IHC), immunofluorescence (IF), Western blot (WB), cell culture and treatments, quantitative RT‒PCR (qPCR), primary murine hepatic stellate cell (HSC) isolation, primary murine HSC and macrophage Raw264.7 co-culture, and flow cytometry (FCM).

## Results

### Reduction of liver fibrosis by preventive pharmacological CCR2 inhibition with CVC

RNA sequencing was performed in control and CCl_4_-induced fibrotic murine livers (Supporting Figure S1A). The top DEGs and KEGG pathway analysis indicated that *Ccr2* and the PI3K-Akt, JAK-STAT, and NFκB signaling pathways were significantly activated in fibrotic livers (Supporting Figure S2A-B). The upregulation of CCR2 was also confirmed in murine and human fibrotic livers by WB (Supporting Figure S2C-D).

**Figure 1:**
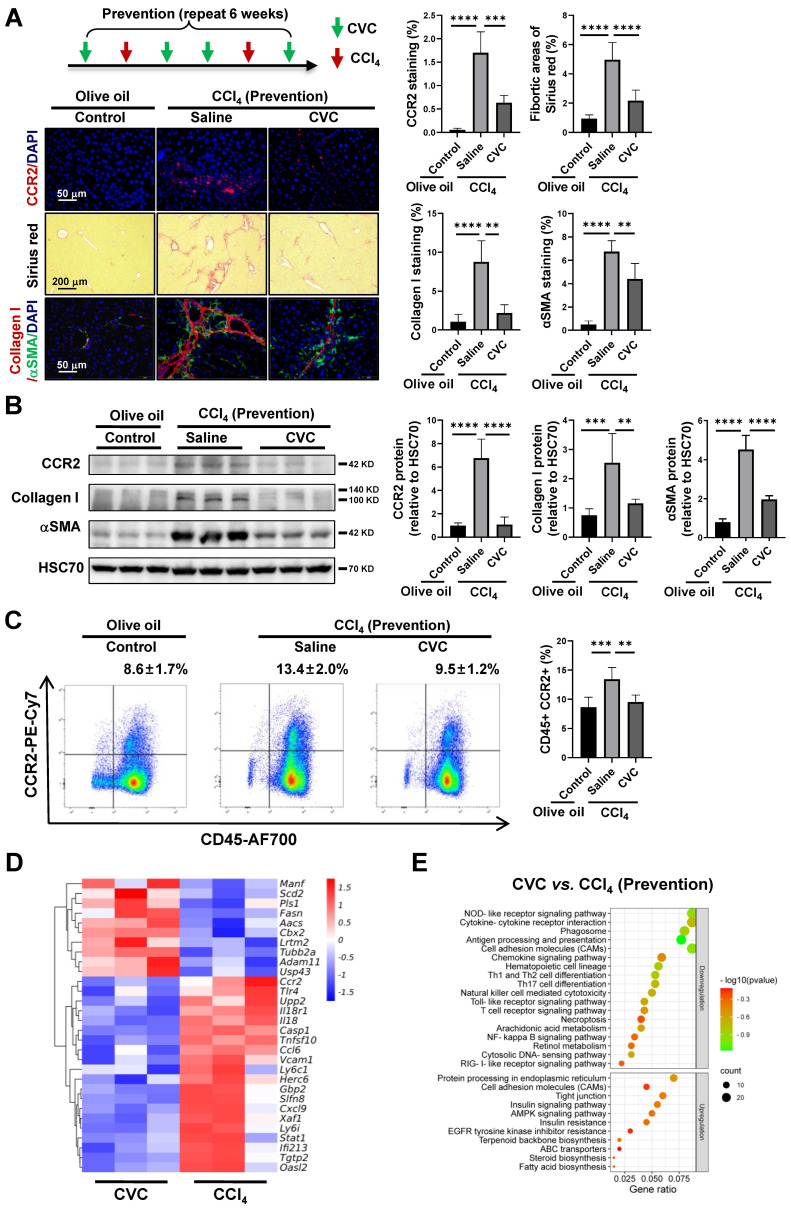
** Reduction of liver fibrosis by preventive pharmacological CCR2 inhibition with CVC. (A-C)** In the prevention experiment, wild-type mice were administered 2 dosages of CCl_4_ in addition to either 4 dosages of saline (CCl_4_ group) or CVC (CVC group) per week for 6 weeks. Mice in the olive oil group were i.p. injected with olive oil for 6 weeks. Liver fibrosis was analyzed by Sirius red staining and IF of collagen I and αSMA (A). The protein levels of CCR2, collagen I, and αSMA were determined by WB (B). The number of CCR2^+^ immune cells in the liver and peripheral blood was quantified by IF and FCM (A, C), n=6/group. **(D-E)** RNA sequencing was performed in murine livers from the CCl_4_ and CVC groups. A heatmap of the top 30 DEGs in the two groups (D) and KEGG pathway analysis of the targeted DEGs (E) are shown. n=3/group. **p<0.01, ***p<0.001, ****p<0.0001.

As CCR2 is upregulated in fibrotic livers, CVC preventive treatment was applied to explore the antifibrotic effects of pharmacological CCR2 inhibition. CVC significantly inhibited the CCR2^+^ immune cells and CCR2 protein levels compared to the CCl_4_ group, as determined by IF and WB (Figure 1A-Β). The increased number of CD45^+^/CCR2^+^ immune cells in the peripheral blood induced by CCl_4_ was also significantly abrogated by CVC (Figure 1C). Consistently, liver fibrosis and hepatic stellate cell (HSC) activation induced by CCl_4_ were also significantly attenuated when preventively treated with CVC, as quantified by decreased fibrotic areas and protein levels of collagen I and αSMA (Figure 1A-B). Meanwhile, the H&E staining of tissues taken from the three groups showed that CVC did not cause damage to other organs, including the heart, kidney, lung, stomach, intestine, and spleen (Supporting Figure S3).

After that, RNA sequencing was applied in the CCl_4_ group and CCl_4_ plus CVC group (CVC group) to explore the disturbed DEGs and signaling pathways with CVC preventive treatment. In total, 757 downregulated and 447 upregulated DEGs were displayed in the CVC group compared to the CCl_4_ group (Supporting Figure S1B). Compared to the CCl_4_ group, proinflammatory and profibrotic genes (*Ccr2*, *Tlr4*, *Il18*, *Ly6c1*, *Herc6*, *Gbp2*, *Slfn8*, *Xaf1*, *Ifi213*) were significantly decreased in the CVC group (Figure 1D). Furthermore, the KEGG signaling pathway analysis of the 757 downregulated DEGs also indicated that associated profibrotic signaling pathways, including the cytokine‒cytokine receptor interaction, PI3K-Akt, JAK-STAT, and NFκB signaling pathways, were inhibited (Figure 1E). In summary, CVC attenuates liver fibrosis by inhibiting CCR2 and CCR2-associated proinflammatory and profibrotic signaling pathways.

### Attenuation of liver fibrosis by therapeutic pharmacological CCR2 inhibition with CVC

**Figure 2:**
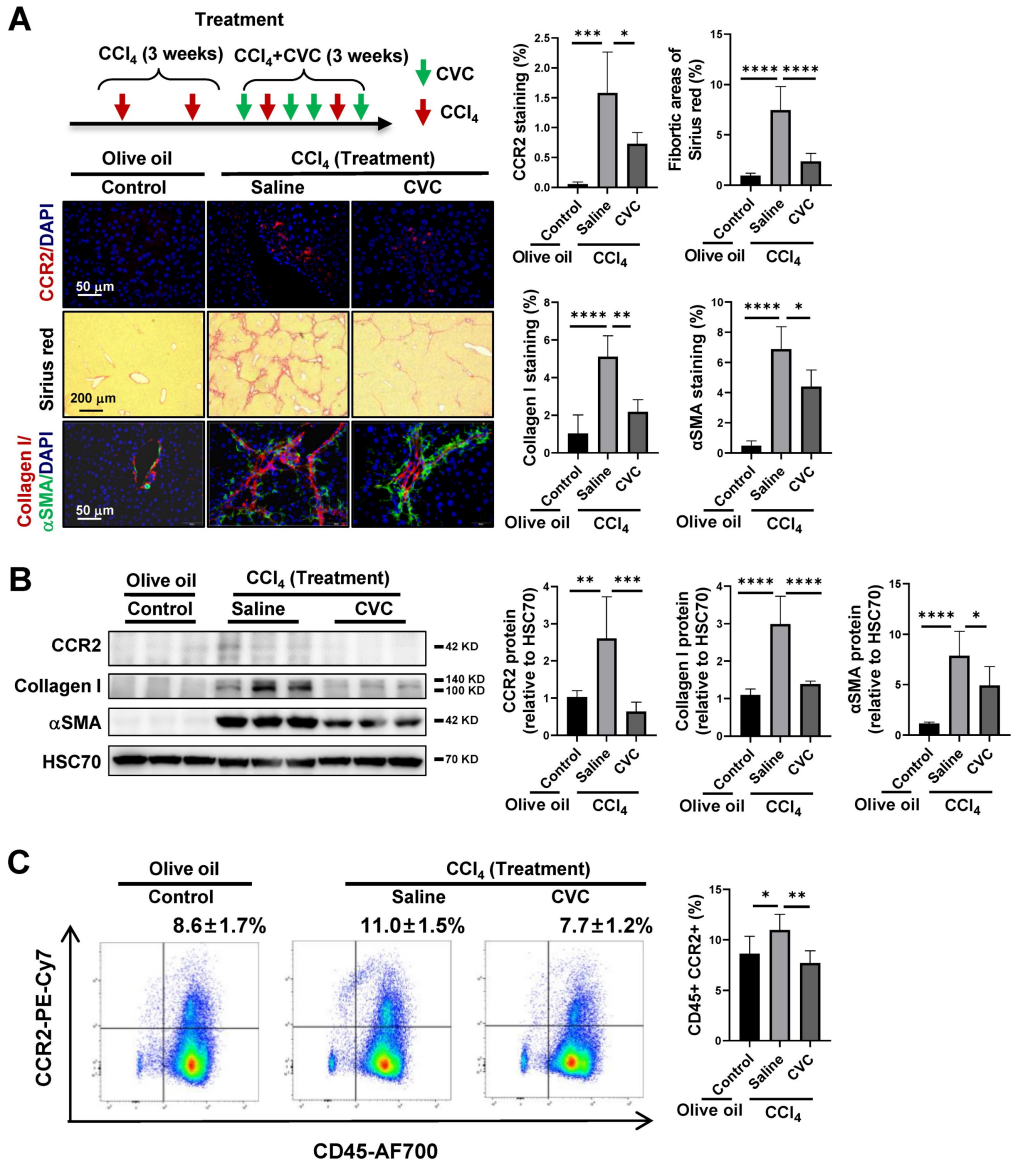
** Attenuation of liver fibrosis by therapeutic pharmacological CCR2 inhibition with CVC. (A-C)** For the treatment experiment, wild-type mice were i.p. injected with CCl_4_ for 3 weeks, in addition to either 4 dosages of saline (CCl_4_ group) or CVC (CVC group) per week for another 3 weeks. Mice in the olive oil group were i.p. injected with olive oil for 6 weeks. Liver fibrosis was analyzed by Sirius red staining and IF of collagen I and αSMA (A). The protein levels of CCR2, collagen I, and αSMA were determined by WB (B). The number of CCR2^+^ immune cells in the liver and peripheral blood was quantified by IF and FCM (A, C), n=6/group. *p<0.05, **p<0.01, ***p<0.001, ****p<0.0001.

To further verify the effects of CVC on existing liver fibrosis, CVC treatment was administered 3 weeks after CCl_4_ injection (Figure 2A). The enhanced protein levels of CCR2 and CCR2^+^ immune cells in the CCl_4_ group were significantly decreased after treatment with CVC (Figure 2A-C). Similar to preventive administration, the ECM accumulation and HSC activation induced by CCl_4_ injection, as quantified by increased fibrotic areas and protein levels of collagen I and αSMA, were also significantly ameliorated by 3 weeks of CVC treatment (Figure 2A-B).

### Restoration of the immune cell landscape by CVC in murine fibrotic liver

**Figure 3:**
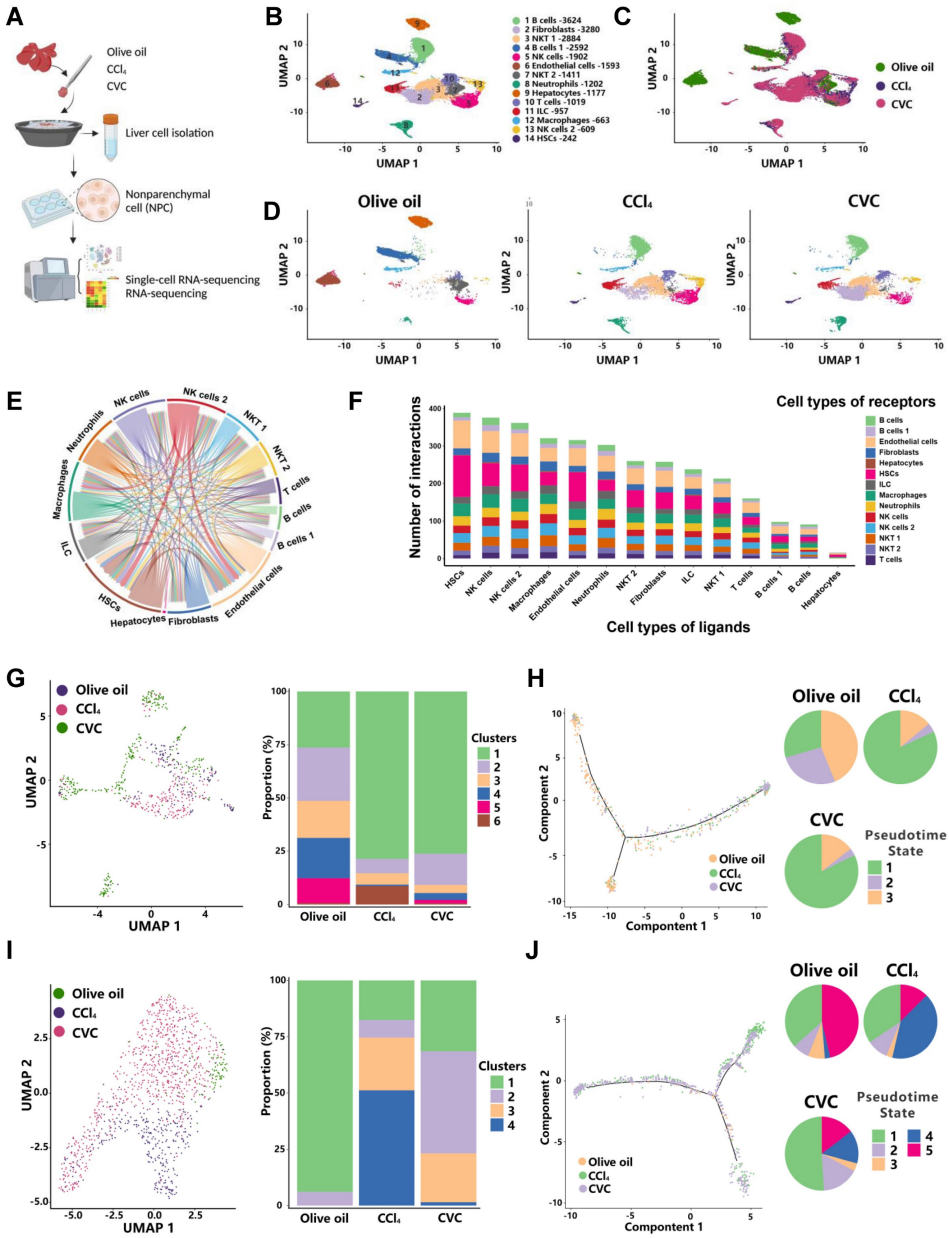
**Restoration of the immune cell landscape by CVC in murine fibrotic liver.** Murine livers from the prevention experiment were subjected to scRNA-seq to determine the immune cell landscape. **(A)** The flow of scRNA-seq analysis. **(B-D)** The relative abundance of 14 cell clusters in the liver (B), the differential distribution of NPCs in the three groups (C), and the changes in the abundance of NPCs in the three individual groups (D) are shown by UMAP plots. **(E-F)** The interactions of 14 cell clusters in the liver (E) and the number of interacting ligand-receptor pairs of 14 cell types (F) are shown. **(G-H)** The ratio changes of 6 macrophage clusters in three groups (G) and pseudotime analysis for 3 pseudotime states of macrophages (H) are displayed. **(I-J)** The ratio changes of 4 neutrophil clusters in three groups (I) and 5 pseudotime states of neutrophils (J) are shown.

Immune cells play a central role in regulating proinflammatory signaling pathways and liver fibrosis.^7^-^11^ The abovementioned results identified that the proinflammatory and profibrotic signaling pathways participate in the antifibrotic effects of CVC. Next, scRNA-seq was applied to nonparenchymal cells (NPCs) to clarify how CVC preventive treatment ameliorates liver fibrosis at the single-cell level (Figure 3A). In total, 22,672 NPCs from murine livers of the 3 groups were assigned to 14 clusters according to the reference marker genes (Figure 3B, Supporting Figure S4). NPCs in the 3 groups showed a differential distribution of cell clusters, including T cells, NK cells, B cells, neutrophils, and macrophages (Figure 3C-D). Cell-to-cell communication analysis was then performed to reveal the interactions among 14 cell clusters. NKT cells (*Ccr5*/*Ccl4*), B cells (*Cd74*/*Mif*), neutrophils (*Ccr1*/*Ccl2*), and macrophages (*Cd74*/*Mif*) mainly interacted with HSCs (Figure 3E-F). Thus, NKT cells, B cells, neutrophils, and macrophages were further divided into subclusters according to the reference marker genes.

Of the 6 clusters of macrophages, increased ratios of clusters 1 and 6 and decreased ratios of clusters 2-5 were observed in the CCl_4_ group compared to the olive oil group (Figure 3G). However, the increased ratio of proinflammatory cluster 6 (*Fscn1*) and decreased ratio of anti-inflammatory cluster 2 (*Xcr1*) were abolished by CVC treatment (Figure 3G, Supporting Figure S5A-B). Additionally, the populations of macrophages in diverse differentiation states were obtained through pseudotime analysis (Figure 3H). An enhanced percentage of pseudotime state 1 and decreased percentages of pseudotime states 2 and 3 were observed in the CCl_4_ group compared to the olive oil group. However, CVC treatment did not restore these changes (Figure 3H).

For the 4 clusters of neutrophils, the decreased ratio of cluster 1 (*Itga4*) and increased ratio of cluster 4 (*Herc6*) induced by CCl_4_ injection were abrogated by CVC treatment (Figure 3I, Supporting Figure S6A-B). Similarly, the increased percentage of pseudotime state 4 induced by CCl_4_ injection was reversed by CVC treatment (Figure 3J). Although some clusters of B cells and T cells were altered between the olive oil group and the CCl_4_ group, there were no significant changes in the 6 clusters of T cells and 10 clusters of B cells between the CCl_4_ group and CVC group (Supporting Figure S7A-D). The increased ratio of macrophages and neutrophils was also validated in the human cirrhotic GEO scRNA-seq dataset GSE136103 (Supporting Figure S8A). Consistently, *FSCN1* and *HERC6* were confirmed as cell markers for macrophages and neutrophils, respectively (Supporting Figure S8B-C). In summary, CVC restores immune cell homeostasis in the murine fibrotic liver by regulating the neutrophil and macrophage landscape. Thus, neutrophils and macrophages were selected as the target cells for further analysis.

### Inhibition of inflammatory macrophage and neutrophil accumulation by CVC

**Figure 4:**
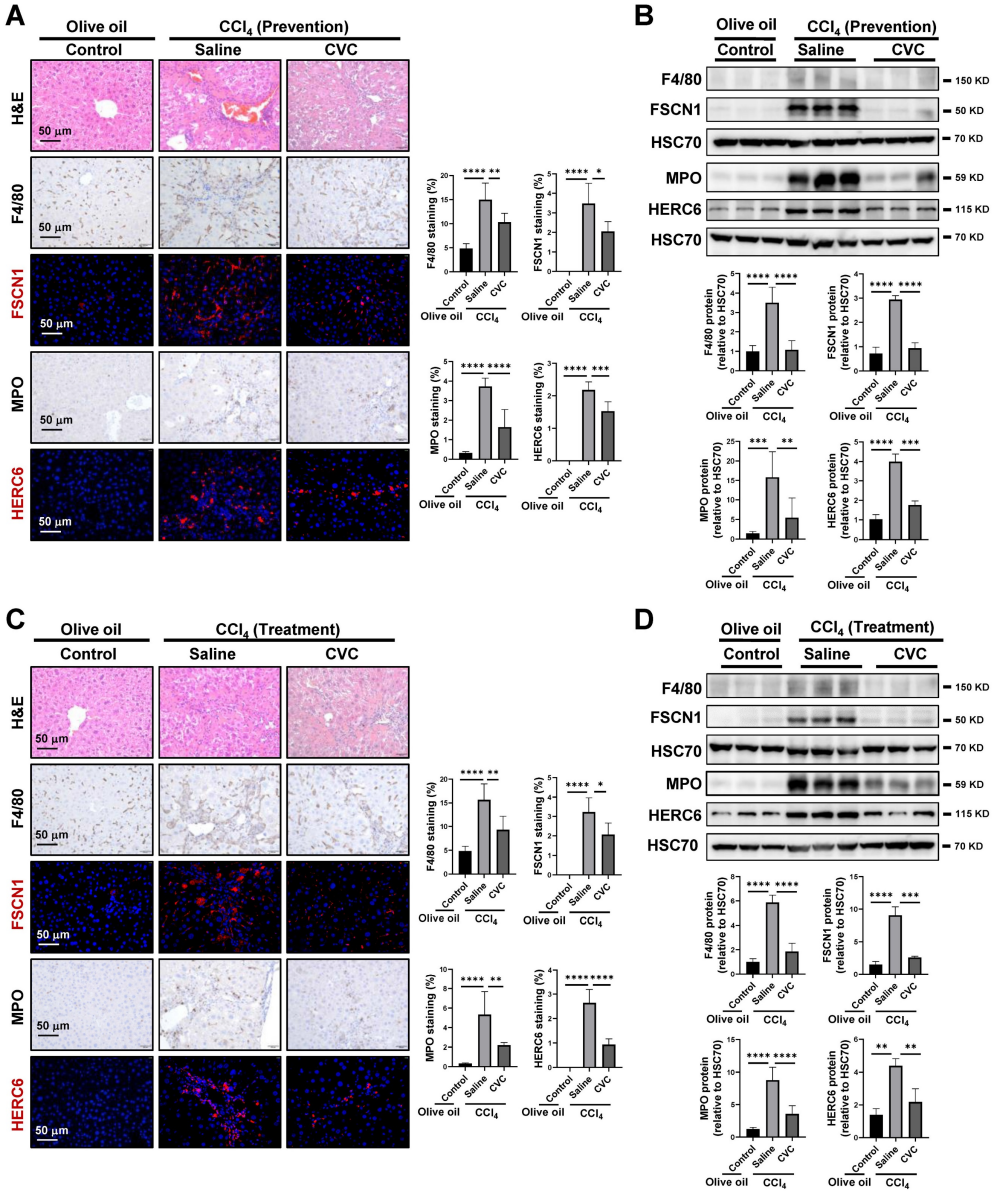
**Inhibition of inflammatory macrophage and neutrophil accumulation by CVC. (A-B)** Inflammatory infiltration was determined in the murine livers of the prevention experiment. H&E staining, IHC of macrophage markers (F4/80, FSCN1), and neutrophil markers (MPO, HERC6, A), and WB for these markers (B) were performed. **(C-D)** Inflammatory infiltration was determined in the murine livers of the treatment experiment. H&E staining, IHC of macrophage markers (F4/80, FSCN1), neutrophil markers (MPO, HERC6, C), and WB for these markers (D) were performed. n=6/group. *p<0.05, **p<0.01, ***p<0.001, ****p<0.0001.

The distributions of macrophages and neutrophils were determined to investigate the disturbed homeostasis of macrophages and neutrophils. In the murine model of CVC prevention, more extensive inflammatory cell infiltration, as determined by H&E staining, was observed in the CCl_4_ group than in the olive oil group (Figure 4A). Consistently, enhanced F4/80^+^ macrophages and myeloperoxidase (MPO)^+^ neutrophils were observed in fibrotic areas of the CCl_4_ group compared to the olive oil group (Figure 4A). FSCN1 and HERC6 are new cell markers for murine proinflammatory macrophage cluster 6 and neutrophil cluster 4 as determined by scRNA-seq (Supporting Figures S5-S6) and human macrophages and neutrophils (Supporting Figures S8B-C). CCl_4_ administration insult increased FSCN1^+^ inflammatory macrophages and HERC6^+^ inflammatory neutrophils compared with the olive oil group. Consistently, the protein levels of F4/80, FSCN1, MPO, and HERC6 were also significantly enhanced in the CCl_4_ group compared to the olive oil group (Figure 4B). However, the abovementioned increases in inflammatory macrophages (F4/80^+^, FSCN1^+^) and inflammatory neutrophils (MPO^+^, HERC6^+^) were remarkably abolished by preventive CVC administration (Figure 4A-B). Similar results were obtained in the CVC treatment murine model. The enhanced hepatic inflammatory macrophage and inflammatory neutrophil accumulation determined by H&E, IF/IHC and WB of F4/80, FSCN1, MPO, and HERC6 in the CCl_4_ group was also significantly abrogated by CVC treatment (Figure 4C-D). In summary, CVC inhibits inflammatory macrophage and neutrophil accumulation in prevention and treatment murine models.

### Attenuation of macrophage and neutrophil recruitment by CCR2 knockout following liver injury

**Figure 5:**
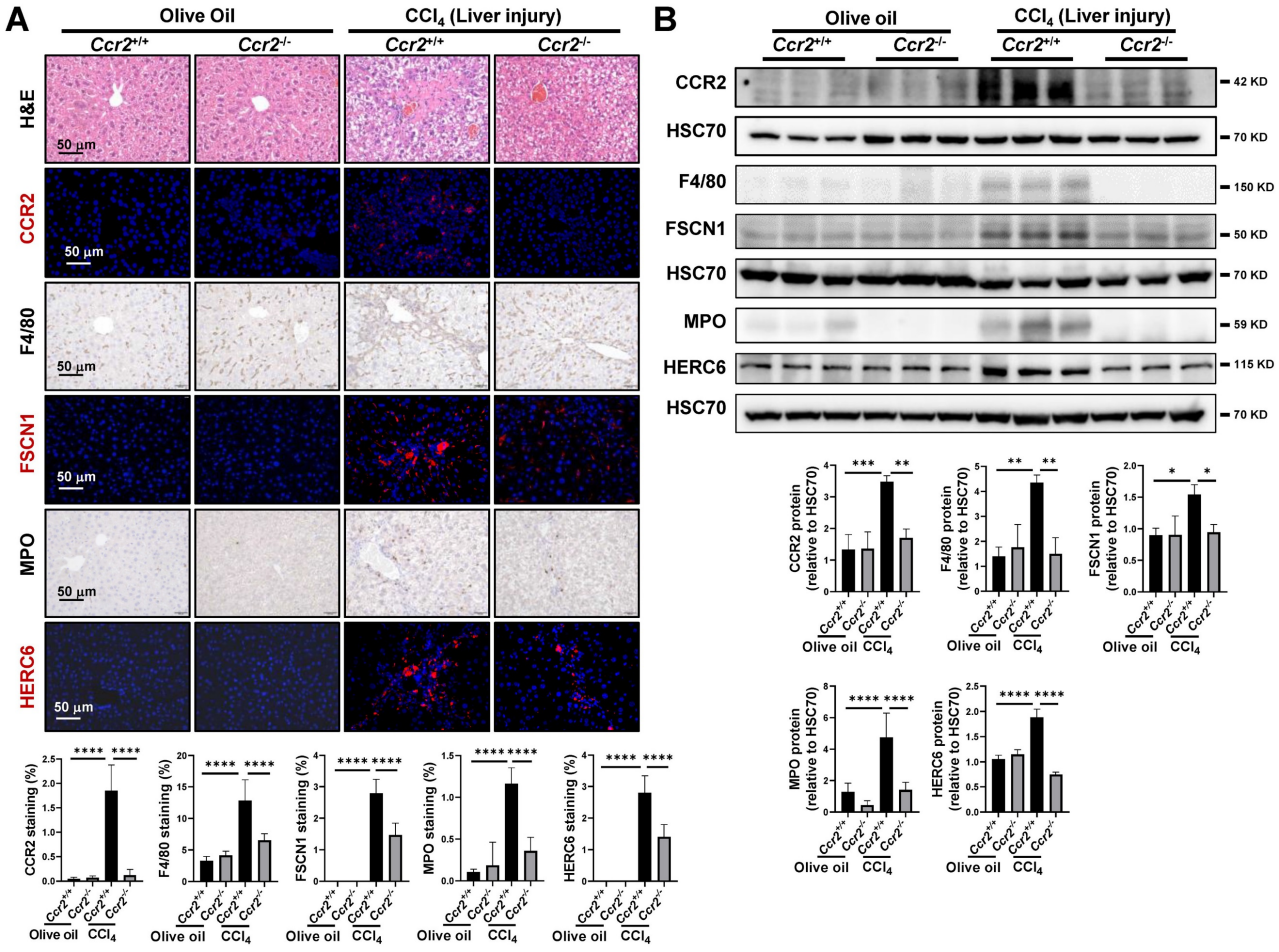
**Attenuation of macrophage and neutrophil recruitment by CCR2 knockout following liver injury. (A-B)** Liver injuries were induced by 2 doses of CCl_4_ in *Ccr2^+/+^* and* Ccr2^-/-^* mice, and mice injected with olive oil were used as controls. H&E staining, IHC and IF of macrophage markers (F4/80, FSCN1), neutrophil markers (MPO, HERC6), and immune cell markers (CCR2, A), and WB for these markers (B) were quantified. n=6/group, *p<0.05, **p<0.01, ****p<0.0001.

In liver fibrosis, macrophage and neutrophil recruitment occur early following liver injury.^18^ Therefore, an acute liver injury mouse model was established in *Ccr2^-/-^* mice to confirm the protective role of CCR2 blockade on macrophage and neutrophil recruitment. CCR2 deletion was confirmed by IF and WB in both olive oil- and CCl_4_-treated mice (Figure 5A-B). The enhanced protein levels of CCR2 and CCR2^+^ immune cells in CCl_4_-treated *Ccr2^+/+^* mice were significantly attenuated after* Ccr2* knockout. (Figure 5A-B). As shown by H&E staining, acute liver injury insult led to immune cells gathering around the portal and central vein areas compared to the olive oil group (Figure 5A). These accumulated immune cells in vascular areas comprised F4/80^+^ macrophages, FSCN1^+^ inflammatory macrophages, MPO^+^ neutrophils, and HERC6^+^ inflammatory neutrophils (Figure 5A). The distribution of these inflammatory macrophages and neutrophils in acute liver injury paralleled the fibrotic septa in liver fibrosis. The accumulation of inflammatory macrophages and neutrophils in the CCl_4_ group compared to the olive oil group was also confirmed by increased protein levels of F4/80, FSCN1, MPO, and HERC6 (Figure 5B). However, the increased inflammatory macrophages and neutrophils following acute liver injury were significantly restored by *Ccr2* knockout (Figure 5A-B). However, comparable levels of the abovementioned immune cells were observed in *Ccr2^+/+^* and *Ccr2^-/-^* mice treated with olive oil (Figure 5A-B). In summary, CCR2 inhibition attenuates inflammatory macrophage and neutrophil recruitment following liver injury.

### The potential mechanism by which CVC restores the immune cell landscape

**Figure 6:**
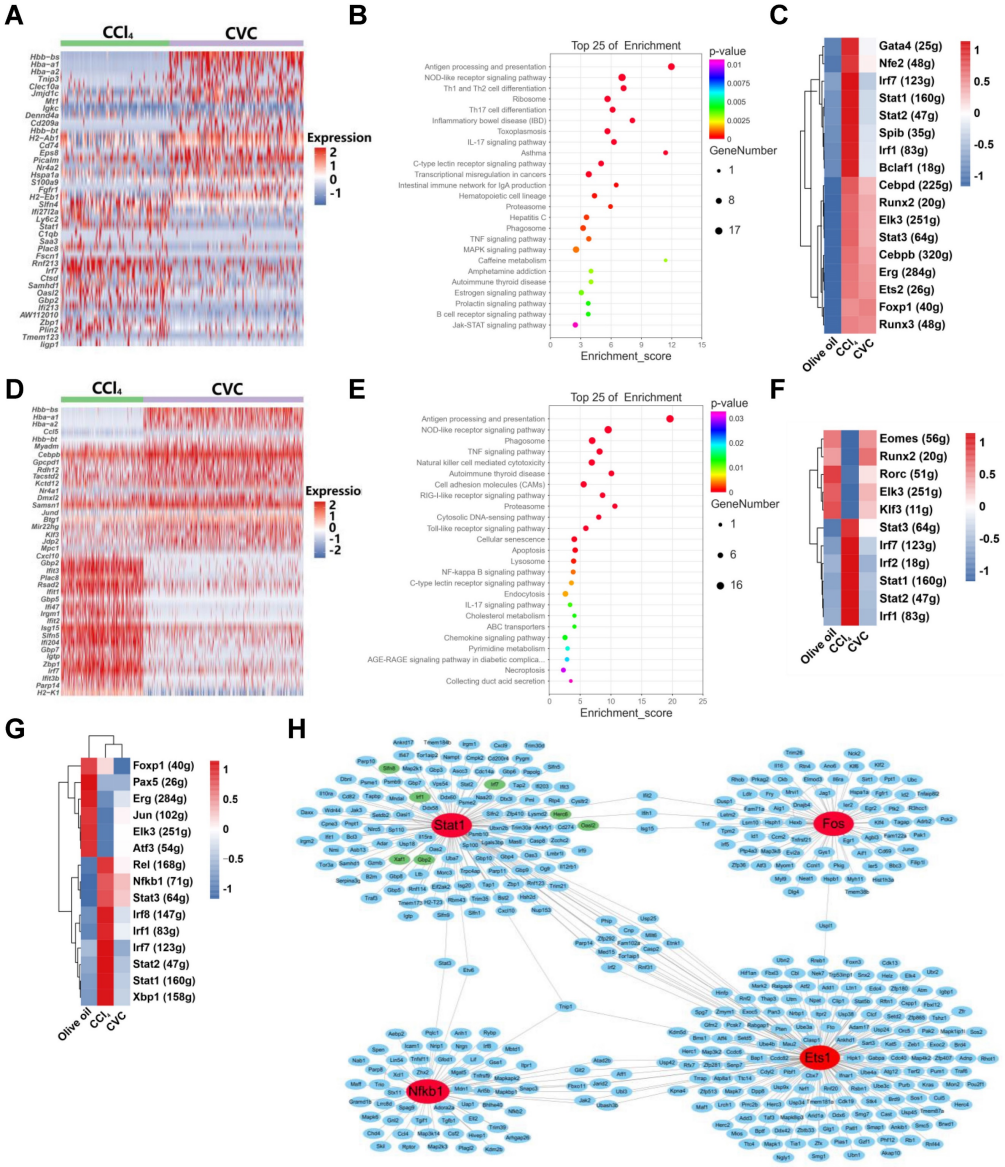
** The potential mechanism by which CVC restores the immune cell landscape.** Murine livers from the prevention experiment were subjected to scRNA-seq to determine the immune cell landscape. **(A-C)** A heatmap of the top 20 downregulated and upregulated DEGs in macrophages of the CCl_4_ and CVC groups (A), KEGG pathway enrichment analysis for all DEGs (B), and a heatmap of RSS in macrophages of the three groups (C) were determined. **(D-F)** A heatmap of the top 20 downregulated and upregulated DEGs in neutrophils of the CCl_4_ and CVC groups (D), KEGG pathway enrichment analysis for all DEGs (E), and a heatmap of RSS in neutrophils of the three groups (F) were displayed. **(G)** The heatmap of RAS for the whole liver in the integrated three groups was shown. **(H)** The transcription factor network analysis in the integrated three groups was revealed.

To further explore the potential mechanism by which CVC improves hepatic inflammatory macrophage and neutrophil accumulation, DEGs, GO enrichment, and KEGG pathway analysis of scRNA-seq were performed. In macrophages, 222 DEGs were found in the CVC group compared to the CCl_4_ group, with the represented downregulated DEGs being *Fscn1, Stat1*, *Xaf1*, *Slfn4*, *Slfn8, Ifi213*, and *Il1β* (Figure 6A). Moreover, the JAK-STAT, NOD-like receptor, TNF, and MAPK signaling pathways were significantly enriched in the KEGG pathway analysis (Figure 6B). The GO enrichment also showed that the cellular response to lipopolysaccharide (LPS) and interferon γ, the immune system process, and DNA-binding transcription factors were crucial in the functions of CVC in restoring the macrophage landscape (Supporting Figure S9A). To understand the deep molecular mechanisms, the gene regulatory networks between transcription factors and DEGs were analyzed with SCENIC software. As shown in the heatmap of the regulon activity score (RAS) for individual macrophages and the integrated three groups, and regulon specificity score (RSS) ranking plots, the activities of transcription factors in the JAK-STAT pathway (*Stat1*, *Stat2*), TNF-NFκB pathway (*Irf1*, *Irf7*), and MAPK-ERK pathway (*Gata4*, *Ets2*) were increased in the CCl_4_ group compared with the olive oil group (Figure 6C, Supporting Fig. S10A-C). However, increased activities of these transcription factors were partly abrogated by CVC treatment, with STAT1 being the most remarkable (Figure 6C, Supporting Figure S10A-C). Presumably, CVC may improve disturbed macrophage homeostasis by regulating *Xaf1*, *Slfn4*, *Slfn8, Ifi213*, and *Il1β via* the JAK-STAT1, TNF-NFκB, and MAPK signaling pathways. The increased *IL1β* in macrophages of the human cirrhotic liver (GSE136103) and enhanced *Xaf1*, *Ifi213*, *Il1β* in macrophages of murine fibrotic livers (GSE157088) were also validated by bioinformatic analysis (Supporting Figure S8D-E).

Similar to macrophages, CVC can also abrogate disturbed neutrophil homeostasis *via* DEGs and related signaling pathways. Compared to the CCl_4_ group, 156 DEGs were found in the CVC group, with the represented downregulated DEGs being *Gbp2*, *Gbp5*, *Gbp7*, and *Herc6* (Figure 6D). In the KEGG pathway analysis, the tumor necrosis factor (TNFα), cytosolic DNA sensing, NOD-like receptor, NFκB, and cellular senescence signaling pathways were significantly enriched (Figure 6E). Meanwhile, the GO enrichment indicated that antigen processing and presentation, cellular response to interferon γ, MHC I protein complex, and TAP binding process were involved in CVC restoring the neutrophil landscape (Supporting Figure S9B). Gene regulatory network analysis was then performed. The enhanced activities of transcription factors in the JAK-STAT pathway (*Stat1*, *Stat2*, *Stat3*) and TNF-NFκB pathway (*Irf1*, *Irf2*, *Irf7*) induced by CCl_4_ were restored by CVC treatment (Figure 6F, Supporting Figure S10D-F). Similarly, the gene regulatory network analysis in the whole liver also indicated that these pathways induced by CCl_4_ were partly abrogated by CVC treatment (Figure 6G, Supporting Figure S10G-I). Moreover, general transcription factor network analysis also indicated that STAT1, NFκB1, ETS1, and FOS can regulate groups of proinflammatory genes (Figure 6H).

In summary, CVC may ameliorate inflammatory macrophage and neutrophil accumulation and liver fibrosis *via* the JAK-STAT1, TNF-NFκB, and MAPK signaling pathways.

### Suppression of STAT1/NFκB/ERK signaling pathway by CVC treatment in murine liver and macrophages

**Figure 7:**
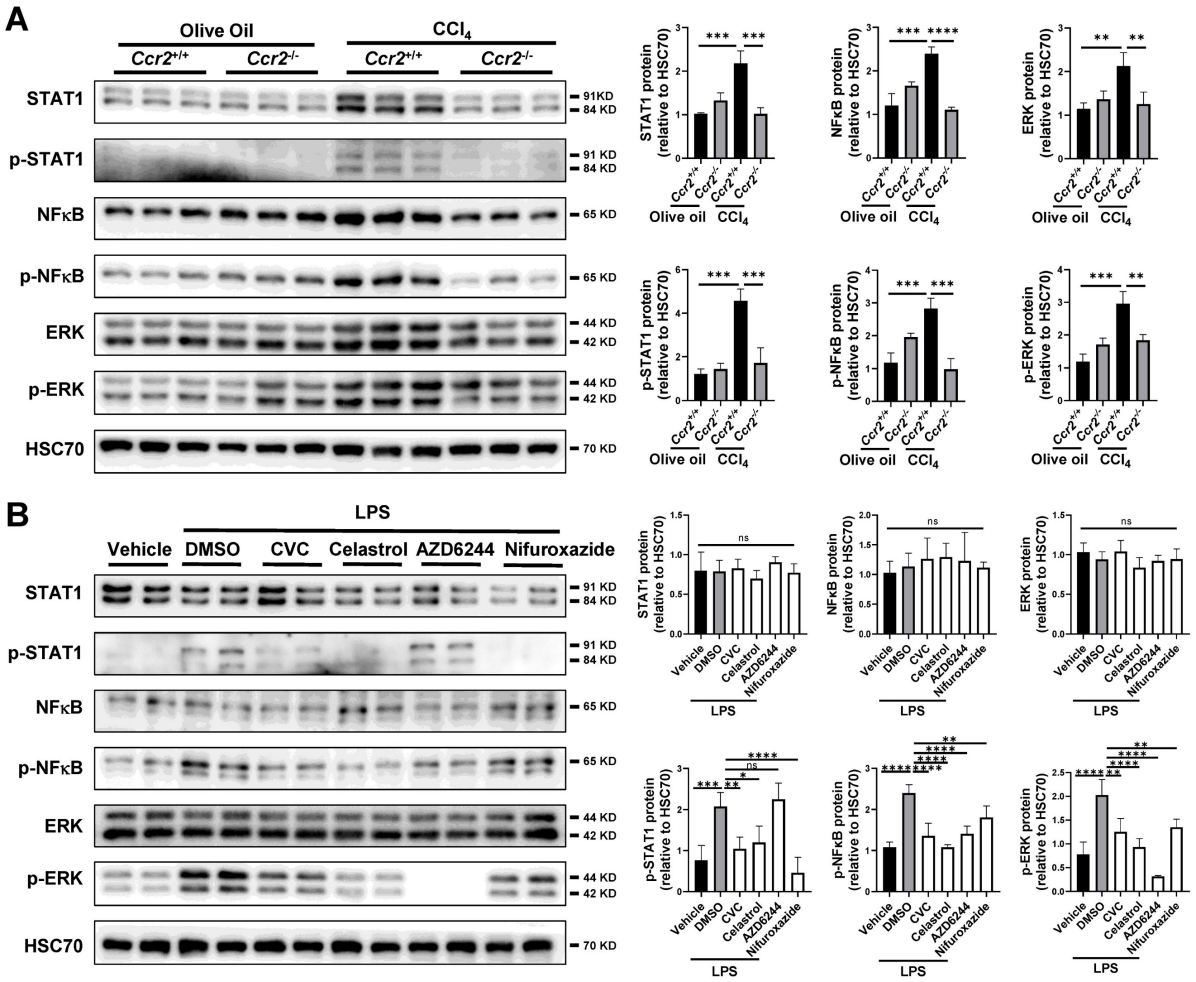
**Suppression of STAT1/NFκB/ERK signaling pathway by CVC treatment in murine liver and macrophages. (A)** *Ccr2^+/+^* or* Ccr2^-/-^* mice were injected with 2 doses of CCl_4_ or olive oil. Protein levels of STAT1, p-STAT1, NFκB, p-NFκB, ERK, and p-ERK were analyzed by WB. N=6/group. **(B)** Raw264.7 murine macrophages were treated with DSMO, CVC, the STAT1 inhibitor nifuroxazide, the NFκB inhibitor celastrol, and the MEK inhibitor AZD6244 for 2 hours followed by stimulation with LPS for another 6 hours. WB was applied to determine the protein levels of STAT1, p-STAT1, NFκB, p-NFκB, ERK, and p-ERK (B). n=3/group. *p<0.05, **p<0.01, ***p<0.001, ****p<0.0001, ns, not significant.

The profibrotic signaling pathways, including the JAK-STAT, TNF-NFκB, and MAPK-ERK signaling pathways, were inhibited by CVC in both scRNA-seq and bulk RNA sequencing. Therefore, we examined the interconnections between CCR2 and these signaling pathways in the murine liver. Compared to those in the olive oil group, the protein levels of phosphorylated STAT1 (p-STAT1), phosphorylated NFκB-p65 (p-NFκB), and phosphorylated ERK (p-ERK) were significantly increased in the CCl_4_-induced liver injury group (Figure 7A). However, the increased p-STAT1, p-NFκB, and p-ERK levels in injured livers were remarkably inhibited by *Ccr2* knockout (Figure 7A).

To explore the molecular interconnective effects of CVC on these signaling pathways, the Raw264.7 murine macrophage lineage was applied* in vitro*. LPS is a typical stimulator of inflammatory macrophages. Compared with vehicle treatment, LPS stimulation significantly increased the protein levels of p-STAT1, p-NFκB, and p-ERK (Figure 7B). The enhanced protein levels of p-STAT1, p-NFκB, and p-ERK were remarkably inhibited in CVC-treated cells (Figure 7B). Furthermore, treatment with the STAT1 inhibitor nifuroxazide, NFκB inhibitor celastrol, and MEK inhibitor AZD6244 also reduced the protein levels of p-STAT1, p-NFκB, and p-ERK, respectively (Figure 7B). These results suggest that the blockade of CCR2 may attenuate inflammatory macrophage and neutrophil accumulation and liver fibrosis by inactivating the JAK-STAT, TNF-NFκB, and MAPK-ERK signaling pathways.

**Figure 8:**
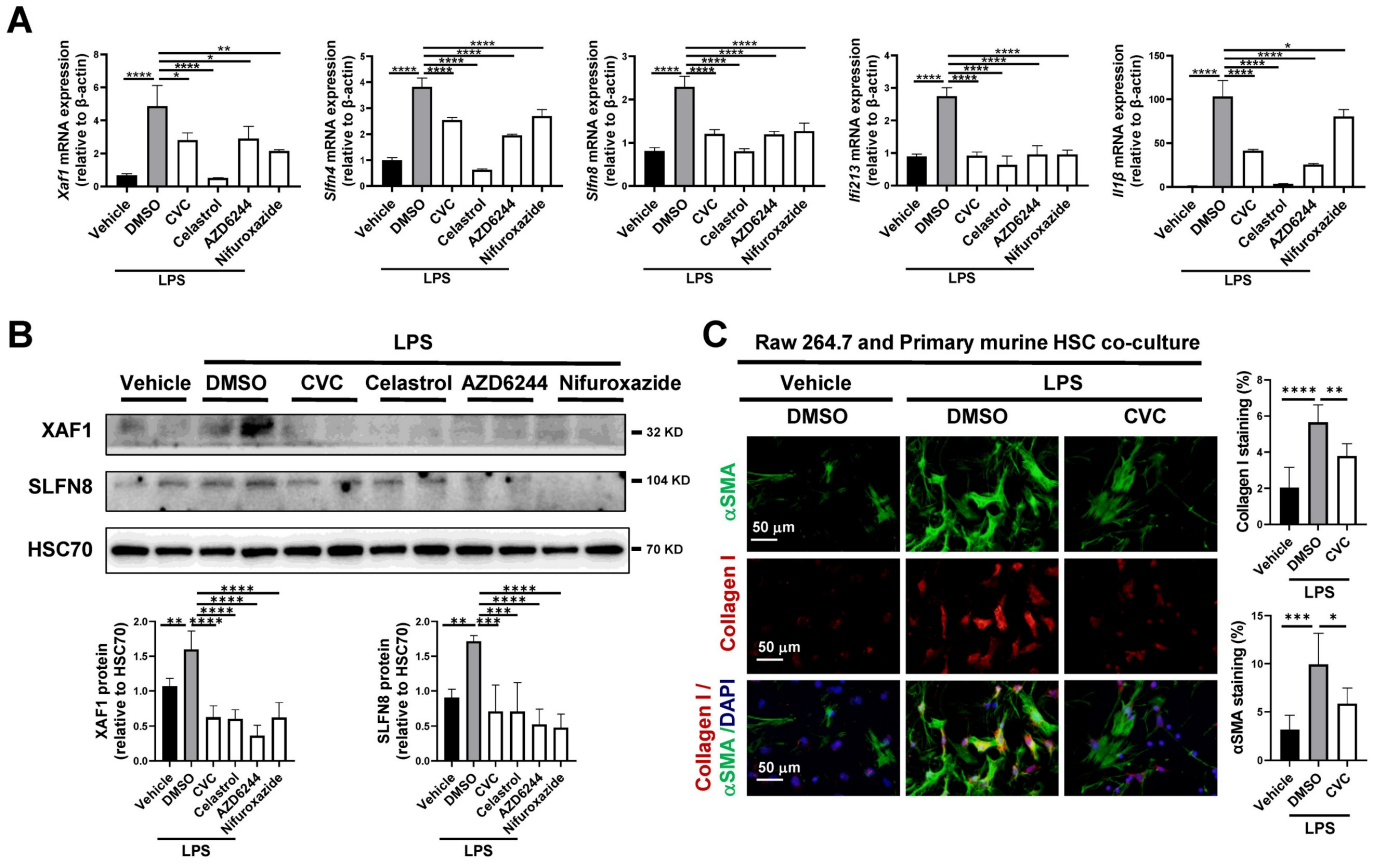
** (A-B)** The mRNA levels of* Xaf1*, *Slfn4*, *Slfn8, Ifi213*, and *Il1β* were analyzed by qPCR (A). The protein levels of XAF1 and SLFN8 were quantified by WB (B). **(C)** Raw264.7 murine macrophages were treated with LPS followed by co-cultured with primary murine HSCs for 48 hours. HSC activation was detected by IF of αSMA and Collagen I. n=3/group, *p<0.05, **p<0.01, ***p<0.001, ****p<0.0001.

In scRNA-seq of macrophages, *Xaf1*, *Slfn4*, *Slfn8, Ifi213*, and *Il1β* were suppressed by CVC. We next verified whether CVC could inhibit the expression of these genes* via* the JAK-STAT1, TNF-NFκB, and MAPK-ERK signaling pathways in Raw264.7 murine macrophages. As expected, the mRNA levels of *Xaf1*, *Slfn4*, *Slfn8, Ifi213*, and *Il1β* in macrophages were significantly elevated under LPS stimulation compared to vehicle-treated cells (Figure 8A). However, these elevated genes were remarkably downregulated by CVC treatment (Figure 8A). Additionally, the enhanced mRNA levels of *Xaf1*, *Slfn4*, *Slfn8, Ifi213*, and *Il1β* induced by LPS were also inhibited by the STAT1 inhibitor nifuroxazide, NFκB inhibitor celastrol, and MEK inhibitor AZD6244 (Figure 8A). Consistently, the increased protein levels of XAF1 and SLFN8 induced by LPS were also inhibited by the STAT1, NFκB and MEK inhibitors (Figure 8B).

A transwell assay was then performed to validate the antifibrotic effect of macrophage CCR2 inhibition with CVC on HSC activation. Raw264.7 murine macrophages were plated into the top well with corresponding treatments, and primary murine HSCs were plated into the bottom of the transwell apparatus. LPS-treated macrophages increased HSC activation, as determined by IF of αSMA and Collagen I in HSCs. However, this HSC activation was abrogated when CVC was added to macrophages (Figure 8C). In summary, CVC inhibits liver fibrosis by repressing gene transcription *via* the CCR2-STAT1/NFκB/ERK signaling pathway.

### Insufficient CVC in regulating intracellular signaling in human fibroblasts and macrophages

The use of CVC in mouse models appears controversial with recent clinical data. Thus, we validated the effects of CVC on intracellular signaling in human macrophage (THP-1) and HSC (LX2) cell lines. In human THP-1 macrophages, LPS stimulation significantly increased the protein levels of p-STAT1, p-NFκB, and p-ERK (Supporting Figure S11A). However, only p-NFκB was significantly suppressed by CVC, while p-ERK was even increased by CVC compared with LPS-treated THP-1 cells (Supporting Figure S11A). Furthermore, no significant changes in the protein levels of p-STAT1, p-NFκB, and p-ERK were observed among the vehicle, LPS, and LPS+CVC groups in LX2 human HSC cell lines (Supporting Figure S12A). In addition, the increased *IL1β* induced by LPS was not abrogated by CVC in either THP-1 or LX2 cells (Supporting Figure S11B, Supporting Figure S12B). In summary, the controversial results of CVC in mouse and human clinical data might be attributed to insufficient regulation of intracellular signaling in human fibroblasts and macrophages.

## Discussion

**Figure 9:**
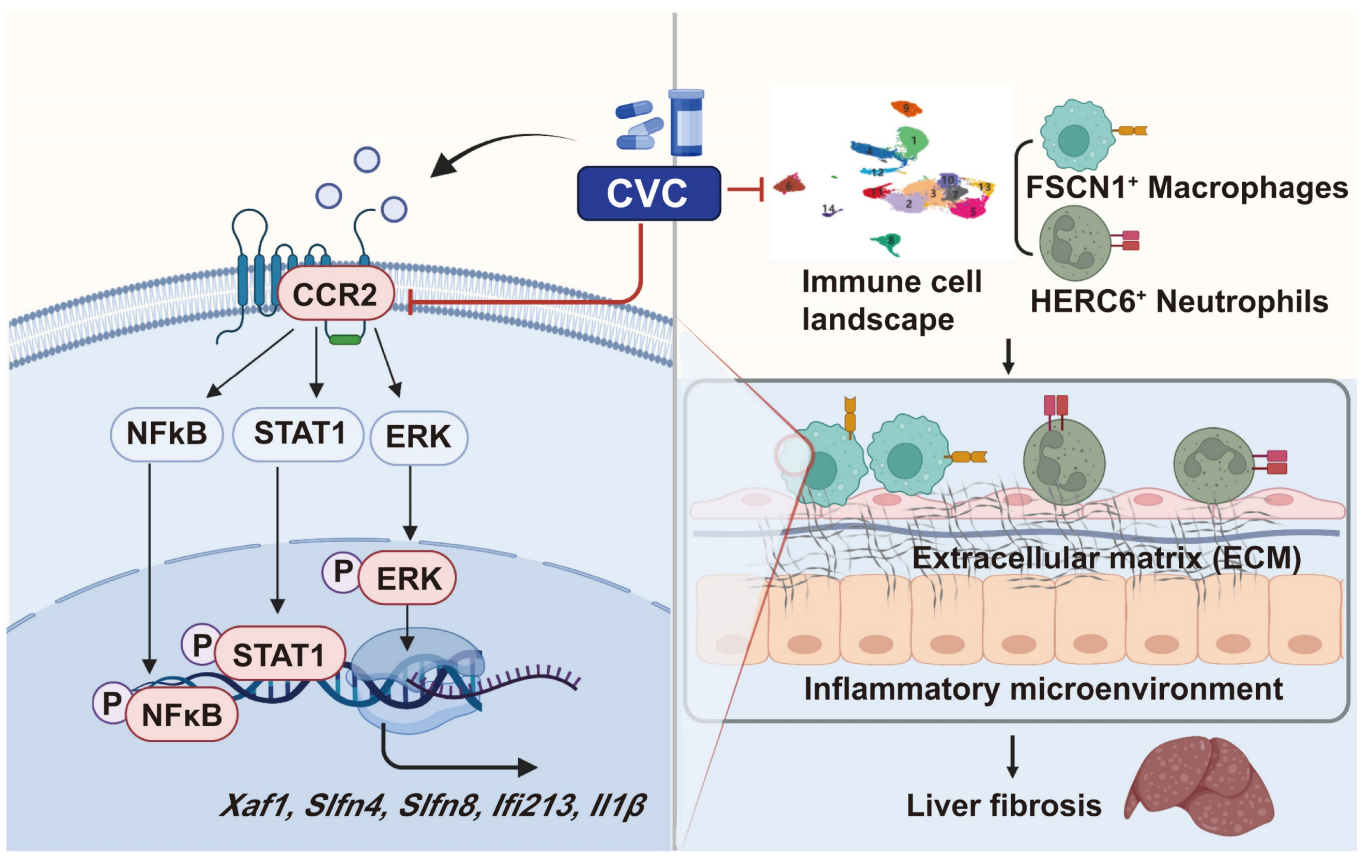
** Proposed mechanisms.** CVC alleviates liver fibrosis by inhibiting hepatic infiltration of FSCN1^+^ macrophages and HERC6^+^ neutrophils. CVC can transcriptionally inhibit *Xaf1*, *Slfn4*, *Slfn8, Ifi213*, and *Il1β* by inactivating the STAT1, NFκB, and ERK signaling pathways.

Chronic inflammation induced by immune cells activates HSCs, leads to ECM deposition, reconstructs hepatic lobules, and eventually promotes liver fibrosis.^5^, ^13^, ^32^, ^33^ Although many clinical practices and basic research have been performed, effective antifibrotic drugs remain deficient. The pharmacological inhibition of CCR2 with CVC has recently been used for the clinical treatment of NASH.^25^ However, the beneficial effects of CVC on human fibrosis were questioned in a recent phase III clinical trial.^29^ Investigations that explore the cellular and molecular mechanisms of CVC in liver fibrosis may help to explain the controversial clinical and experimental data. Unlike previously reported antifibrotic effects of CVC in NASH and ALD, we discovered a new role for CVC in ameliorating CCl_4_-induced liver fibrosis by restoring the proinflammatory microenvironment at the single-cell level. We demonstrated a new cellular mechanism by which CVC inhibits ECM accumulation in liver fibrosis by suppressing the hepatic accumulation of inflammatory FSCN1^+^ macrophages and HERC6^+^ neutrophils. Furthermore, we verified a novel molecular mechanism by which CVC reduces profibrotic gene transcription by inactivating the STAT1/NFκB/ERK signaling pathways (Figure 9). Collectively, this study clarified the new role and novel mechanism of CVC in attenuating liver fibrosis by restoring the proinflammatory microenvironment.

CCR2 is a marker gene depicting immunocyte infiltration and fibrotic severity in preclinical research.^19^, ^20^, ^34^ Activated CCR2 causes M1-polarized macrophage recruitment and ECM-producing HSC activation, ultimately leading to liver fibrosis.^12^, ^21^, ^35^ Consistently, we verified the upregulation of CCR2 in murine and human fibrotic livers. Recent studies have demonstrated that pharmacological inhibition of CCR2 by CVC decreases hepatic steatosis and fibrosis 15, 25, 26 Furthermore, CCR2 inhibition by CVC can improve insulin resistance and steatohepatitis in NASH patients.^27^, ^28^ Whether CVC can also improve liver fibrosis in other models remains unknown. We verified the antifibrotic effects of CVC by reducing ECM accumulation on CCl_4_-induced liver fibrosis from both prevention and treatment perspectives. Our results indicate that pharmacological inhibition of CCR2 by CVC might become a key novel strategy for liver cirrhosis therapy without causing other organ injuries.

During liver injury, classical monocytes accumulate in the sinusoid and differentiate into CCR2^+^ (Ly6C^high^) macrophages, eventually exacerbating liver fibrosis^12^, ^36^. Consistently, our previous study explored whether LSEC-mediated aberrant angiocrine signals can recruit CCR2^+^ MDM accumulation in CCl_4_-induced liver fibrosis.^12^ In addition, CCR2 is also expressed in T/NK cells and neutrophils, and neutrophils also lead to CCR2-mediated macrophage infiltration and HSC activation^37^, ^38^. Previous studies on CVC treatment focused on inhibiting the recruitment of Ly6C^high^ monocytes in murine NASH and ALD models.^15^, ^25^ However, whether other clusters of immune cells also contribute to the antifibrotic effects of CVC remains unclear. Using scRNA-seq, we identified the immune cell landscape of how CVC improves liver fibrosis by restoring the proinflammatory microenvironment. CVC predominantly inhibited the hepatic accumulation of inflammatory FSCN1^+^ macrophages and HERC6^+^ neutrophils in prevention and treatment administration. Moreover, *Ccr2* knockout also ameliorated the abovementioned inflammatory macrophage and neutrophil infiltration during early liver injury. As CVC is a dual CCR2/CCR5 inhibitor, it is presumable that CVC mainly inhibits inflammatory macrophage and neutrophil accumulation *via* CCR2. Using scRNA-seq and experimental validation, we have clarified that the antifibrotic effect of CVC is attributed to abrogating the proinflammatory microenvironment.

Once activated by the chemokine ligand CCL2, CCR2 can stimulate multiple downstream signaling pathways in cancers, including the PI3K-Akt, RAC-GTPase, and PKC signaling pathways.^39^ However, the downstream signaling pathways and profibrotic genes activated by CCR2 in the context of liver fibrosis have not been fully explored. Using scRNA-seq, bulk RNA sequencing, and experimental validation *in vivo* and *in vitro*, we identified that the JAK-STAT1, TNF-NFκB, and MAPK-ERK signaling pathways contribute to the antifibrotic effects of CVC. For profibrotic genes activated by CCR2, CVC can reverse alcohol-related increases in hepatic TNFα, IL1β, IL-6, and CCL2 in an ALD murine model.^25^ In this study, we have displayed a hallmark of CVC-mediated profibrotic genes during liver fibrosis. *Xaf1*, *Slfn4*, *Slfn8, Ifi213*, and *Il1β* are the common hub profibrotic genes downregulated by CVC both *in vivo* and *in vitro*. CVC can inhibit the transcription of these profibrotic hub genes by inactivating the STAT1, NFκB, and ERK signaling pathways in the murine liver and macrophages. However, except for *Il1β*, which has well-known functions during liver fibrosis,^40^, ^41^ *Xaf1*, *Slfn4*, *Slfn8,* and* Ifi213* are profibrotic genes with unknown functions. Thus, further investigations are urgently needed to explore how inhibition of *Xaf1*, *Slfn4*, *Slfn8,* and* Ifi213* by CVC can attenuate liver fibrosis. In contrast, CVC is insufficient in regulating these intracellular signaling pathways in human fibroblasts and macrophages, which may explain the controversial clinical and experimental data.

In conclusion, we clarify the cellular and molecular mechanisms of the antifibrotic effects of CVC by restoring the proinflammatory microenvironment. CVC ameliorates ECM deposits and liver fibrosis by suppressing the hepatic accumulation of inflammatory FSCN1^+^ macrophages and HERC6^+^ neutrophils. The antifibrotic effects of CVC have been attributed to transcriptionally inhibiting hub profibrotic genes by inactivating the STAT1/NFκB/ERK signaling pathways. These results elucidate the antifibrotic strategy with CVC for the clinical treatment of liver cirrhosis.

## Supplementary Material

Supplementary methods, figures and tables.

## Abbreviations

αSMA: α-smooth muscle actin
ALD: alcohol-related liver disease
CCR2: C-C motif chemokine receptor 2
CCL2: C-C motif chemokine ligand 2
CVC: cenicriviroc
CCl_4_: carbon tetrachloride
DEGs: differentially expressed genes
ECM: extracellular matrix
ERK: extracellular regulated protein kinases
FCM: flow cytometry
FSCN1: fascin actin-bundling protein 1
HERC6: HECT and RLD domain containing E3 ubiquitin protein ligase family member 6
H&E: hematoxylin and eosin
HSC: hepatic stellate cell
IHC: immunohistochemistry
IF: immunofluorescence
*Ifi213*: Interferon-activated gene 213
*Il1β*: Interleukin 1 beta
LSECs: liver sinusoidal endothelial cells
LPS: lipopolysaccharide
MEK: mitogen-activated extracellular signal-regulated kinase
MDMs: monocyte-derived macrophages
MPO: myeloperoxidase
NASH: nonalcoholic steatohepatitis
NFκB: nuclear factor-κB
NPCs: nonparenchymal cells
p-STAT1: phosphorylated-STAT1
p-ERK: phosphorylated-ERK
p-NFκB: phosphorylated-nuclear factor-κB
qPCR: quantitative real-time polymerase chain reaction
RAS: regulon activity score
RSS: regulon specificity
scRNA-seq: Single-cell RNA sequencing
*Slfn4*: Schlafen 4
*Slfn8*: Schlafen 8
*Xaf1*: XIAP-associated factor 1
STAT1: signal transducer and activator of transcription 1
WB: Western blot
