# Supplementary material for: C-C motif chemokine receptor 2 inhibition reduces liver fibrosis by restoring the immune cell landscape
Source: Int J Biol Sci. 2023 May 8;19(8):2572–87. doi: 10.7150/ijbs.83530 (PMC10197881; doi:10.7150/ijbs.83530)
Supplement: Supplementary file 1 — Supplementary methods, figures and tables. [file ijbsv19p2572s1.pdf]

Supporting Materials

C-C motif chemokine receptor 2 inhibition reduces liver fibrosis by restoring the immune cell landscape

Yangkun Guo[1,2#], Chong Zhao[1,2#], Wenting Dai[1,2#], Bowen Wang[1,2,3], Enjiang Lai[1,2], Yang Xiao[1,2], Chengwei Tang[1,2], Zhiyin Huang[2], Jinhang Gao*[1,2]

[1]*Lab of Gastroenterology and Hepatology, State Key Laboratory of Biotherapy, West China Hospital, Sichuan University, Chengdu, China.*

[2]*Department of Gastroenterology; West China Hospital, Sichuan University, Chengdu, China.*

[3]*Department of Gastroenterology; General Hospital of Tibet Military Command, Lhasa, China*

[#]Yangkun Guo, Chong Zhao, and Wenting Dai contributed equally to this study.

Correspondence:

Jinhang Gao, M.D., Ph.D., Professor

Lab of Gastroenterology and Hepatology, West China Hospital, Sichuan University, NO. 1, 4th Keyuan Road, Chengdu, 610041, China.

Tel: 86-28-85104011

E-mail: Gao.jinhang@scu.edu.cn or Gao.jinhang@qq.com.

Supporting Methods

Single-cell RNA sequencing (scRNA-seq) and bioinformatics analysis

scRNA-seq and bioinformatics analysis were performed by Shanghai OE Biotech Inc. (Shanghai, China). The murine livers were washed with precooled RPMI-1640 plus bovine serum albumin nutrient medium and minced to approximately 0.5 mm^2 cubic pieces on ice. The obtained tissues were digested with the enzyme at 37°C followed by filtration through 40 μm cell strainers. Then, the cell suspension was centrifuged at 50 g for 5 minutes to remove hepatocytes. The cell pellet was resuspended in red blood cell lysis buffer to remove erythrocytes. After pouring out the supernatant, the cell pellets were resuspended again with 100 μL substrate for further cell concentration and viability calculation using the Luna cell counter. The prepared single-cell suspension was detected by high-throughput sequencing, and raw data in FASTQ format were obtained. These transcriptome results were quantified by CellRanger software (version 5.0.0) based on cellular barcodes and unique molecular identifiers (UMIs). Primary quality control was performed with the CellRanger and the Seurat package (version 3.1.1) was utilized to further select high-qualified cells according to the standard indices of nUMI, nGene, and percent.mito. Moreover, potential doublets were discarded using DoubletFinder software (version 2.0.2). Then, highly variable genes (HVGs) were visualized by FindVariableGenes in the Seurat package, and principal component analysis (PCA) and uniform manifold approximation and projection (UMAP) plots were used to identify the cell clusters. The marker genes of these cell clusters and subclusters were determined by FindAllMarkers of Seurat. A criterion of p-value <0.05

and fold change >1.5 was set to screen differentially expressed genes (DEGs), which were visualized in KEGG pathway enrichment analysis with the R package. To obtain the pseudotime trajectory of gene expression, the differentialGeneText function of the Monocle2 package was applied, and their corresponding cells were displayed with reduced dimensions. The ligand-receptor-based cellphoneDB database was utilized to reveal the interactions between cell clusters. The gene regulatory networks were analyzed using SCENIC software (Hirst Courtney, UK). Cytoscape was applied to characterize the interactions between transcription factors and genes. The raw scRNA-seq data have been deposited in the NCBI Gene Expression Omnibus (GSE218496).

Bioinformatic analysis of the public GEO dataset

We performed clustering and differential gene expression analyses of published raw data of single-cell RNA sequencing (GSE136103) by R version 4.2.2. In this dataset, single-cell transcriptomic data were obtained from 5 healthy and 5 cirrhotic livers.[1] All of the liver-resident cells were grouped into 11 clusters. The heatmaps of gene markers of the 11 clusters and violin plots were produced using the Seurat package coupled with the ggplot2 package.

Gene expression analyses of published RNA sequencing data (GSE157088) were also performed by R. In this dataset, RNA sequencing was performed in primary macrophages (F4/80^+ $CD45^+$ UV^- PI^-) that were isolated from wild-type mice treated with one dose of olive oil (n=3) or CCl_4 (1 mL/kg, n=3).[2]

RNA sequencing

RNA sequencing and bioinformatics analysis were performed by Novogene (Beijing,

China). Total RNA was extracted from liver cell samples using an RNeasy Mini Kit and assessed with a Nanodrop. Prepared RNA samples were fragmented in a fragmentation buffer, and reverse transcription was performed with a cDNA synthesis kit. A PCR kit was utilized to amplify the obtained cDNA followed by visualization with Illumina software. The low-qualified data were deleted by FastQC software, and this selected sequence was matched to the original sites according to the reference genome. Then, differential expression analysis was performed by the R package. The results were visualized in a heatmap and KEGG plots. The raw RNA-seq data have been deposited in the NCBI Gene Expression Omnibus (GSE218497).

Hematoxylin and eosin (H&E) staining and Sirius red staining

Paraffin-embedded liver sections were rehydrated with xylene and graded ethanol dilutions. Sections were then stained with hematoxylin for 10 seconds, followed by eosin staining for 1 minute. Sirius red was utilized for 30 minutes. Sections were dehydrated, and the liver fibrotic area was observed with a light microscope (CX41, Olympus Corporation, Tokyo, Japan).

Immunohistochemistry (IHC) and immunofluorescence (IF)

For IHC, antigen retrieval of the deparaffinized sections was performed in sodium citrate buffer (10 mM, pH=6.0) at a boiling temperature for 15 minutes. Sections were blocked with donkey serum at 37°C for 1 hour and then incubated with primary antibodies overnight at 4°C. After that, sections were incubated with corresponding secondary antibodies followed by an ABC reagent. Sections were detected with 3,3'-diaminobenzidine (DAB, ZSGB-BIO). Sections were counterstained with hematoxylin

and captured under a histology microscope (CX41).

For IF, paraffin-embedded sections were incubated with 0.5% Triton X-100, followed by blocking with donkey serum at 37°C. Primary antibodies against different species were applied to liver tissue sections overnight at 4°C, and appropriate fluorescent dye-conjugated secondary antibodies were then applied for 1 hour. Next, liver sections were stained with 4',6-diamidino-2-phenylindole (DAPI) solution, and the fluorescent signals were detected with fluorescence microscopy (BX53, Olympus Corporation). The primary antibodies used in the study are listed in Supporting Table S2.

Western blot (WB)

Liver tissues and Raw264.7 murine macrophages were lysed with a protease and phosphatase inhibitor cocktail containing lysis buffer. The liver tissue homogenate was centrifuged at 12000 rpm to extract the protein in the supernatant. The protein concentration was determined by bicinchoninic acid (BCA) assay. Next, 20-50 μg of protein was added to each well of the prepared SDS–PAGE gel. The electrophoretic protein bands were obtained by gel electrophoresis at 100 V for 90 minutes and then transferred onto a PVDF membrane. The membrane was blocked with 5% nonfat dry milk, followed by incubation with primary antibodies overnight at 4°C. After rewarming and washing with 1× TBST, the membrane was incubated with horseradish peroxidase (HRP)-labeled secondary antibodies for 1 hour. Then, the signals of the bands were visualized and quantified using ImageJ software with a chemiluminescence detection kit. The HSC70 of mice was regarded as the loading control. The primary

antibodies used in the study are listed in Supporting Table S2.

Cell culture and treatments

The Raw264.7 murine macrophages were purchased from the Cell Bank of Type Culture Collection of Chinese Academy of Sciences China (Shanghai, China) and verified by STR profiling. Raw264.7 macrophages were cultured in Dulbecco's modified Eagle's medium (DMEM) containing 10% fetal bovine serum (FBS) and 1% penicillin–streptomycin solution in humid air with 5% CO_2 at 37°C. Cells were starved with 1% FBS for 2 hours and then treated with dimethyl sulfoxide (DMSO), CVC (1 μM, Selleck #S8512), the STAT1 inhibitor nifuroxazide (1 μM, Selleck #S1491), the NFκB inhibitor celastrol (1 μM, Selleck #S1290), or the MEK inhibitor AZD6244 (1 μM, Selleck #S1008). Afterward, macrophages were stimulated with LPS (1 μg/mL, Invitrogen #tlrl-3pelps) for another 6 hours. Cells were collected for further experiments.

Quantitative RT–PCR (qPCR)

The RNeasy Mini Kit (Foregene #RE-03014) was utilized to extract total RNA from Raw264.7 macrophages. Then, 500 μL of buffer RL1 was added to the lysed cells and transferred to a DNA-cleaning column to remove DNA pollution. After that, the supernatant was collected and mixed with buffer RL2. RNA in this solution was well attached by an RNA-only column followed by washing with buffer RW1 and RW2. The RNA-only column was then washed with 65°C preheated RNase-Free water to collect purified RNA solution, whose OD 260/280 ratio was detected by Nanodrop. A total of 20 μL of the mixture system was utilized to perform reverse transcription, and the

obtained cDNA was further used for qPCR using SYBR Green (Bimake #B21202). The forward and reverse primers designed by Primer Premier 5.0 are listed in Supporting Table S3.

Primary murine hepatic stellate cell (mHSC) isolation

Primary mHSCs were isolated with the perfusion method as previously described.[3] Under anesthesia, the murine liver was perfused *in situ* with EGTA, proteases (0.4 mg/mL, Roche #25551121), and collagenase 1A (0.5 mg/mL, Sigma–Aldrich) at room temperature. Then, the liver cell suspension was obtained by smashing the digested liver, followed by passing through a 70 μm cell strainer. HSCs were collected with density gradient centrifugation.

Primary mHSCs and Raw264.7 co-culture with a transwell assay

The co-culture system was performed using a 24-well transwell chamber with a permeable membrane of 0.4 μm pore size (Corning #3450). Freshly isolated primary mHSCs were seeded into the lower chamber, and Raw264.7 murine macrophages were seeded into the upper chamber. Macrophage inflammatory polarization was induced by LPS (1 μg/mL), followed by DMSO or CVC. The control Raw264.7 cells were treated with a vehicle. The macrophages in the upper chamber were transferred into the lower chamber 24 hours after administration. After co-cultured for an additional 48 hours, HSC activation was determined by IF of αSMA and Collagen I.

Flow cytometry (FCM)

Peripheral blood was obtained from the right eye of wild-type mice, CCl_4-treated mice, and CCl_4 plus CVC-treated mice, followed by lysing the red blood cells for 15 mins on

ice. The pellet was collected by centrifuging at 300 g for 10 minutes at 4°C and resuspended in BD Horizen solution (BD Bioscience #565388) in PBS for live/dead staining. Then, the cell suspension was pelleted, resuspended in PBS, and blocked with 50 μL Fc block solution for 5 minutes at 4°C. The samples were incubated with the corresponding antibody cocktail at room temperature (CD45 and CCR2, Supporting Table S2), and transferred to a flow tube for flow cytometer analysis. $CCR2^+$ immune cells were identified by double positivity for CD45 and CCR2.

Supporting Tables

Supporting Table S1. Clinical information of patients with liver cirrhosis and normal controls

Group	Age	Gender	Diagnosis	HBV	Sample
**Hepatectomy surgery**					
Normal 1	57	F	Normal	-	Liver tissue
Normal 2	26	F	Normal	-	Liver tissue
Normal 3	42	M	Normal	-	Liver tissue
Normal 4	69	F	Normal	-	Liver tissue
Normal 5	51	F	Normal	-	Liver tissue
Normal 6	49	F	Normal	-	Liver tissue
Normal 7	23	M	Normal	-	Liver tissue
Cirrhosis 1	79	M	Cirrhosis	-	Liver tissue
Cirrhosis 2	54	M	Cirrhosis	+	Liver tissue
Cirrhosis 3	70	M	Cirrhosis	+	Liver tissue
Cirrhosis 4	57	M	Cirrhosis	+	Liver tissue
Cirrhosis 5	39	M	Cirrhosis	-	Liver tissue
Cirrhosis 6	56	M	Cirrhosis	-	Liver tissue
Cirrhosis 7	50	M	Cirrhosis	+	Liver tissue
Cirrhosis 8	58	M	Cirrhosis	+	Liver tissue
Cirrhosis 9	53	M	Cirrhosis	-	Liver tissue
Cirrhosis 10	60	M	Cirrhosis	-	Liver tissue
Cirrhosis 11	69	M	Cirrhosis	-	Liver tissue

Supporting Table S2. Antibody list for immunofluorescence (IF) immunohistochemistry (IHC), flow cytometry (FCM), and Western blot (WB)

Antibody	Application	Dilution	Source	Company and cat no
**αSMA**	IF	1:200	Rabbit	Abcam #124964
	WB	1:2000		
**CCR2**	WB	1:1000	Rabbit	Cell Signaling Technology #12199
	IF	1:100	Rabbit	Abcam #273050
	FCM	1:100	Rat	BioLegend #150612
**CD45**	FCM	1:100	Rat	BD Bioscience #560510
**Collagen I**	IF	1:100	Goat	Southern Biotech #1310-01
	WB	1:1000	Rabbit	Abcam #260043
**ERK**	WB	1:2000	Rabbit	Selleck# A5029
**p-ERK**	WB	1:2000	Rabbit	Selleck# A506
**F4/80**	IHC	1:100	Rabbit	Cell Signaling Technology #70076
	WB	1:1000		
**FSCN1**	IF	1:100	Mouse	Cell Signaling Technology #99978
	WB	1:1000	Rabbit	ABclonal #A1904
**GAPDH**	WB	1:10000	Mouse	ABclonal #AC033
**HERC6**	IF	1:100	Rabbit	Huabio #ER1910-50
	WB	1:1000		
**HSC70**	WB	1:1000	Mouse	Santa Cruz Biotechnology #Sc-7298
**MPO**	IHC	1:200	Rabbit	Abcam #208670
	WB	1:1000		
**NFκB-p65**	WB	1:1000	Rabbit	Cell Signaling Technology #8242T
**p-NFκB-p65**	WB	1:1000	Rabbit	Cell Signaling Technology #3033
**SLFN8**	WB	1:1000	Rabbit	Biorbyt #Orb186088
**STAT1**	WB	1:1000	Rabbit	Cell Signaling Technology #14994
**p-STAT1**	WB	1:1000	Rabbit	Cell Signaling Technology #9167
**XAF1**	WB	1:1000	Rabbit	Cell Signaling Technology #13805

IHC: Immunohistochemistry; WB: Western blot; IF: Immunofluorescence; FCM: Flow cytometry; αSMA: Alpha-smooth muscle actin; CCR2: C-C chemokine receptor 2; ERK: extracellular regulated protein kinases; HSC70: Heat shock cognate protein 70; MPO: Myeloperoxidase; NFκB: nuclear factor-κB; SLFN8, Schlafen 8; STAT1: signal transducer and activator of transcription 1; XAF1: XIAP-associated factor 1.

Supporting Table S3. List of primers for qRT–PCR

Gene	Sequence-forward (5′-3′)	Sequence-reverse (5′-3′)
**Primers for mouse qRT–PCR**		
Ccr2	AATAATCATTTTGTTCTCTGACCAC;<br>AATAATCATTTTGTTCTCTGACCAC	CTGAACTTGTGGCCTTTAC;<br>ACAGCATGAACAATAGCCAAGT
Ifi213	GCCCTCCTCCACTTACCTC	GCCCTCCTCCACTTACCTC
Il1β	TTGAAGTTGACGGACCCC	GTGCTGCTGCGAGATTTG
Snlf4	TTATCCACCTGCGTTCGG	CCTGGTTCCTTGGGTTTTAC
Snlf8	CGTTGACCGTGACTCTTTG	ACACCTTGTCCTTGTCTTTAG
Xaf1	CTGCCTTTGAAGTCTGGG	CTGGAGTTTCTTTTGGTGAG
**Primers for human qRT–PCR**		
IL1β	TTCGAGGCACAAGGCACAA	TTGAAGTTGACGGACCCC

Ccr2: C-C chemokine receptor 2; *Ifi213*: Interferon-activated gene 213; *Il1β*: interleukin 1β; *Snlf8*: Schlafen 8; *Xaf1*: XIAP-associated factor 1.

Supporting Figures

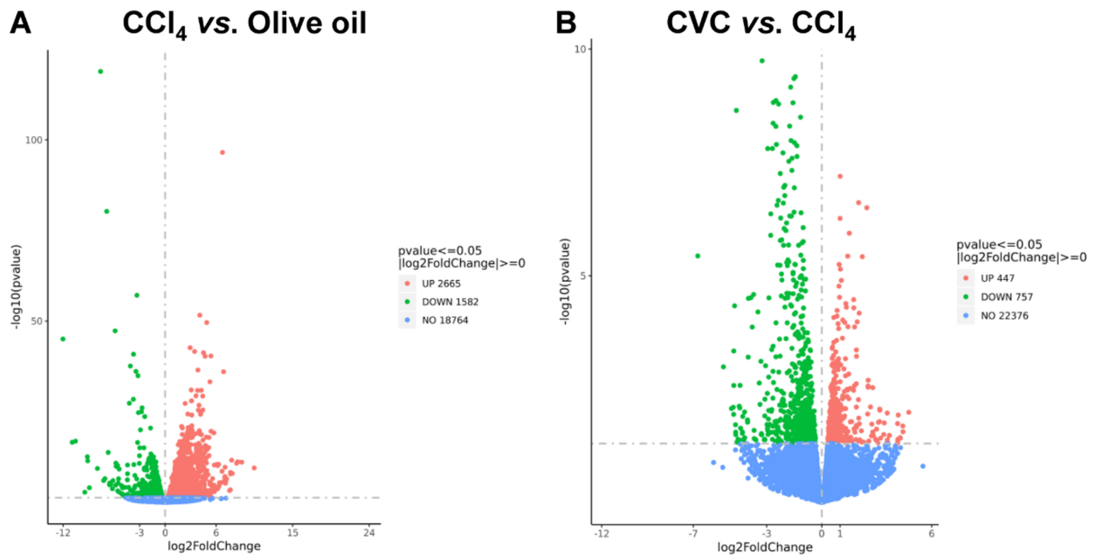

Supporting Figure S1. Volcano plot of DEGs

Upregulated, downregulated, and nonsignificant genes in the CVC group relative to the CCl_4 group are shown by the volcano plot.

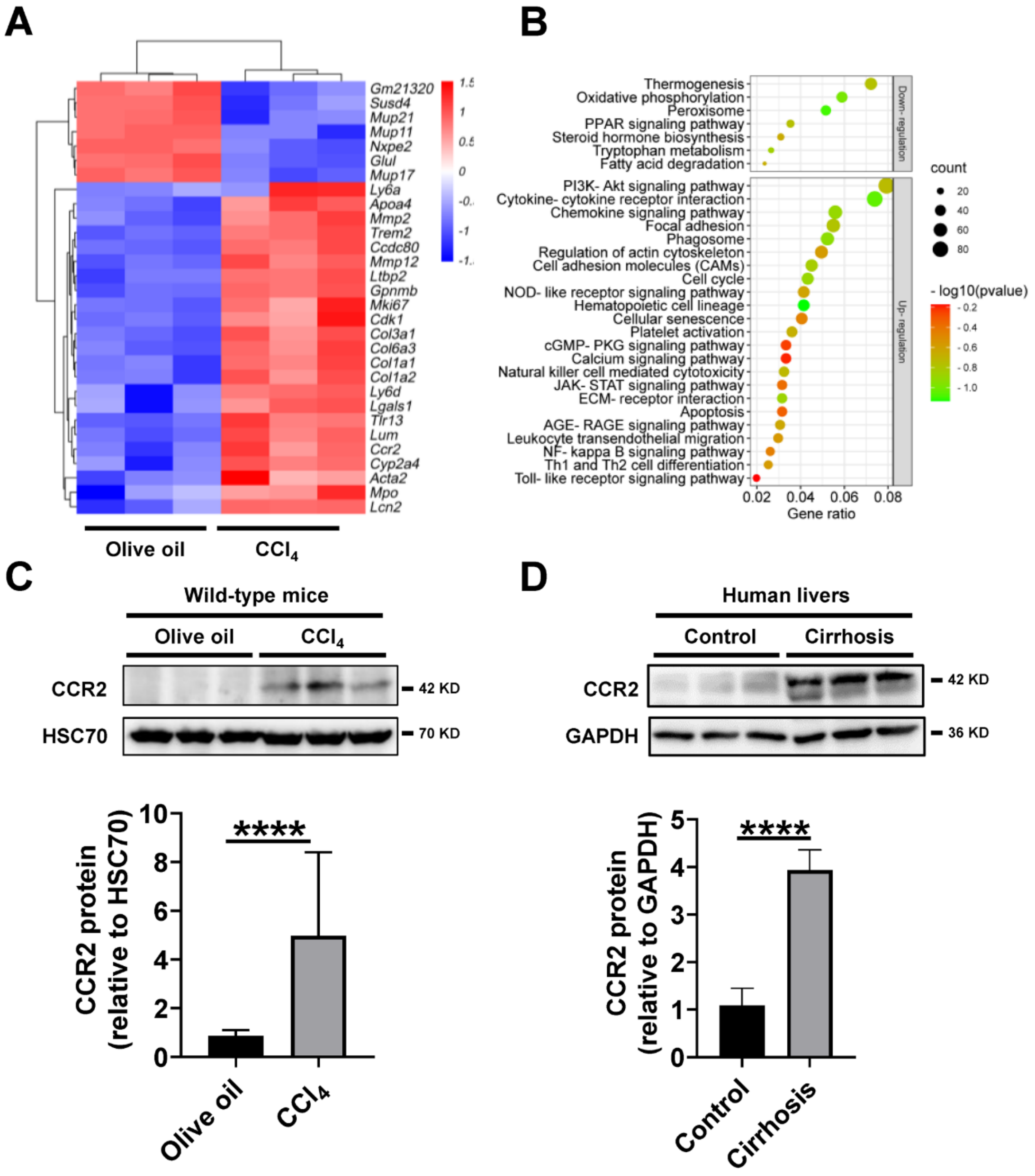

Supporting Figure S2. CCR2 upregulation in fibrotic livers

(A-B) Wild-type mice were i.p. injected with olive oil or CCl_4 for 6 weeks. RNA sequencing was performed to analyze the gene expression patterns. A heatmap of the top 30 DEGs in the two groups (A) and KEGG pathway analysis of these DEGs (B) are shown. n=3/group.

(C) The protein levels of CCR2 in murine control and CCl_4-induced fibrotic livers were determined by WB. n=6/group.

(D) The protein levels of CCR2 in human control (n=7) and cirrhotic livers (n=11) were determined by WB.

****p<0.0001.

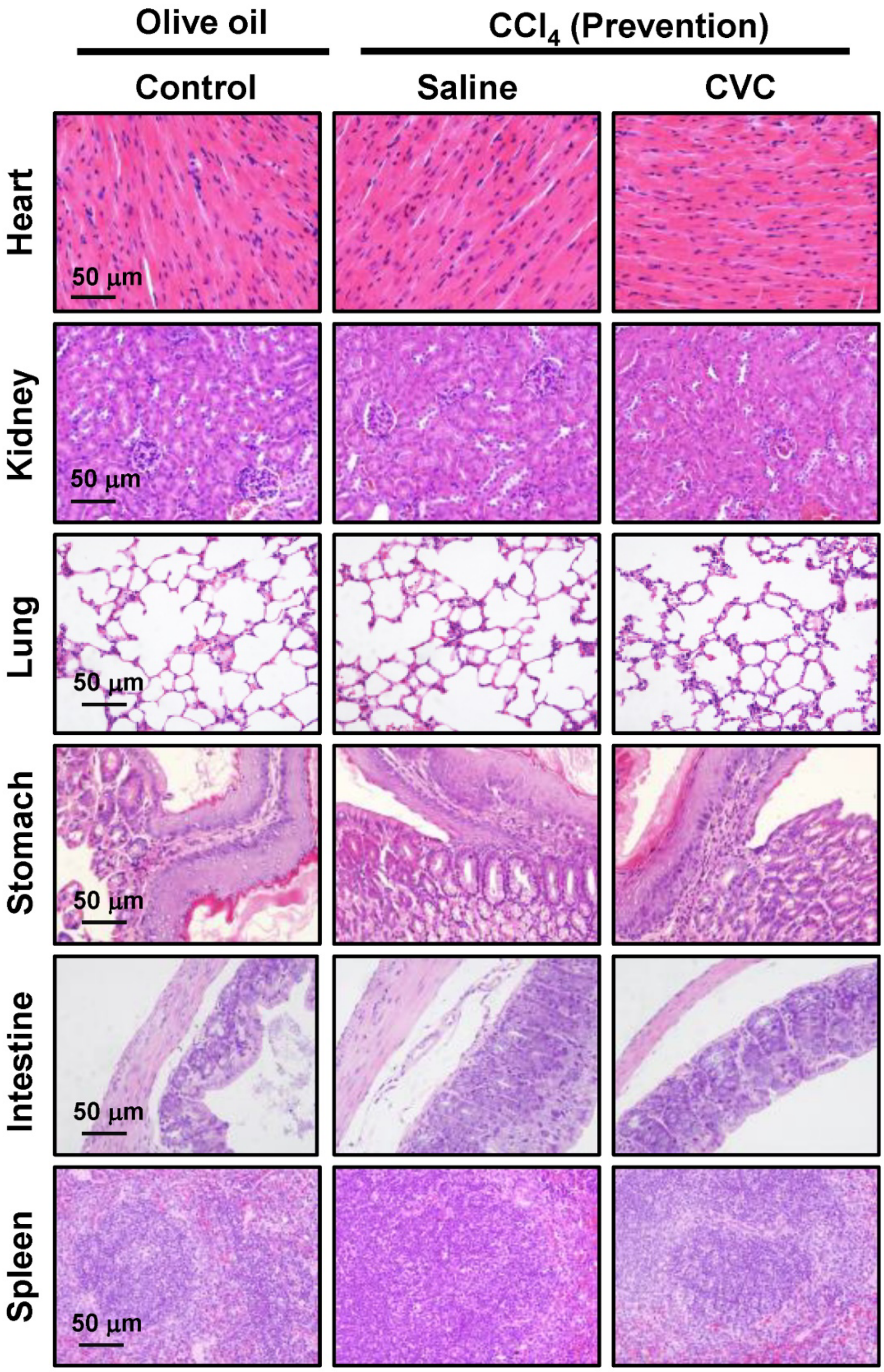

Supporting Figure S3. CVC does not lead to organ injury in murine liver fibrosis

Murine organs of the prevention experiment were subjected to H&E staining.

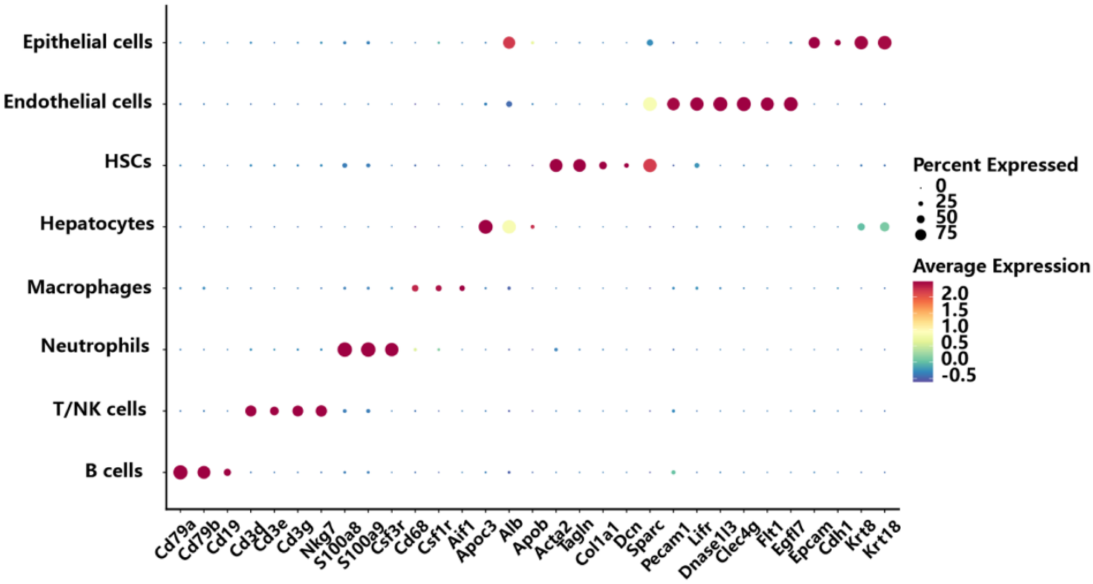

Supporting Figure S4. Cell markers of each cluster

In the scRNA-seq data of the prevention experiment, marker genes were displayed for epithelial cells, endothelial cells, HSCs, hepatocytes, macrophages, neutrophils, T/NK cells, and B cells.

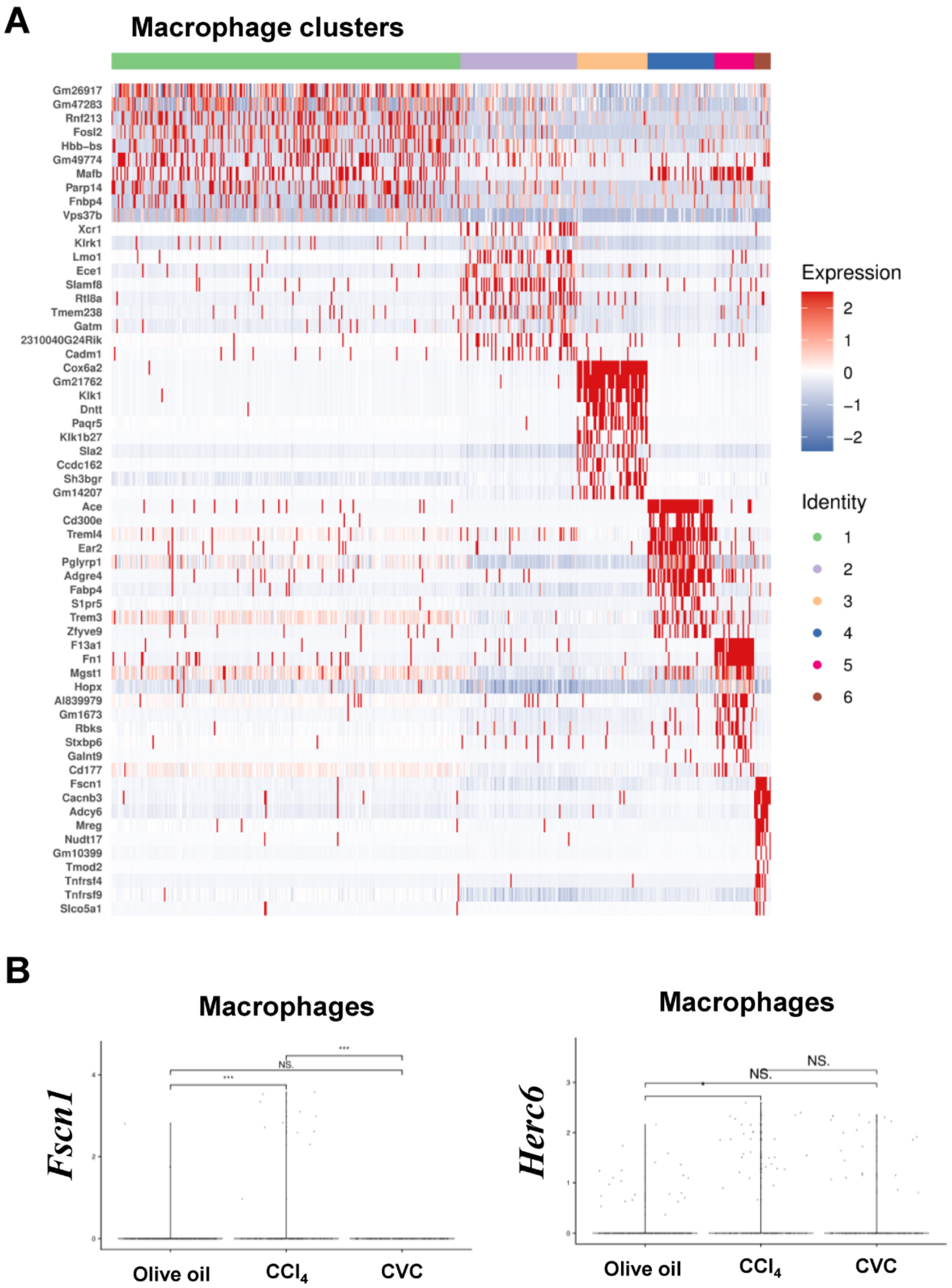

Supporting Figure S5. Marker gene expression of macrophages in scRNA-seq

(A) Expression of the top 10 marker genes in macrophage clusters in scRNA-seq data.

(B) The expressions of *Fscn1* and *Herc6* in macrophages were visualized by violin plots.

**p*<0.05, ****p*<0.001, NS., not significant

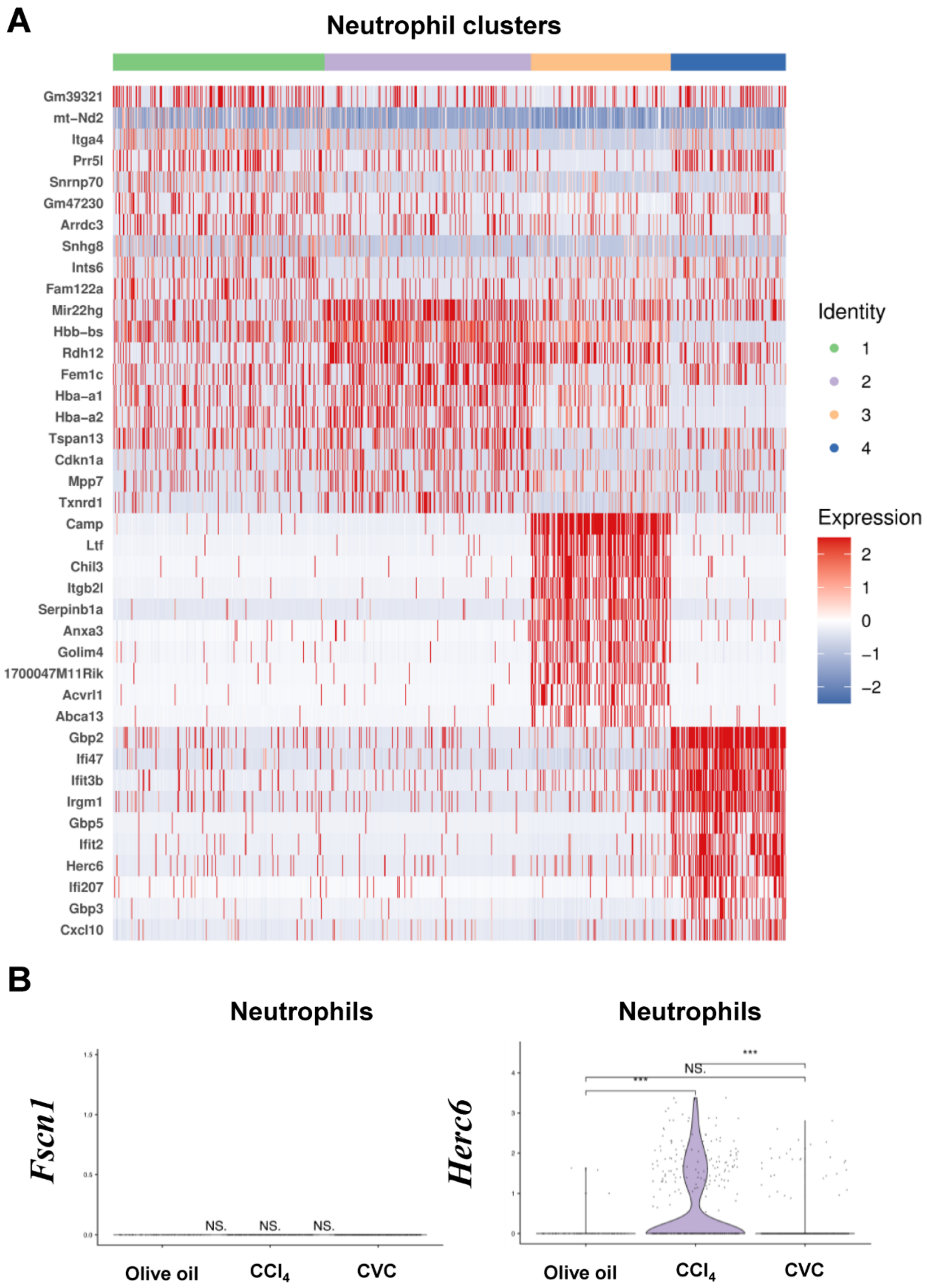

Supporting Figure S6. Marker gene expression of neutrophils in scRNA-seq

(A) Expression of the top 10 marker genes in neutrophil clusters in scRNA-seq data.

(B) The expressions of *Fscn1* and *Herc6* in neutrophils were visualized by violin plots.

***p<0.001, NS., not significant.

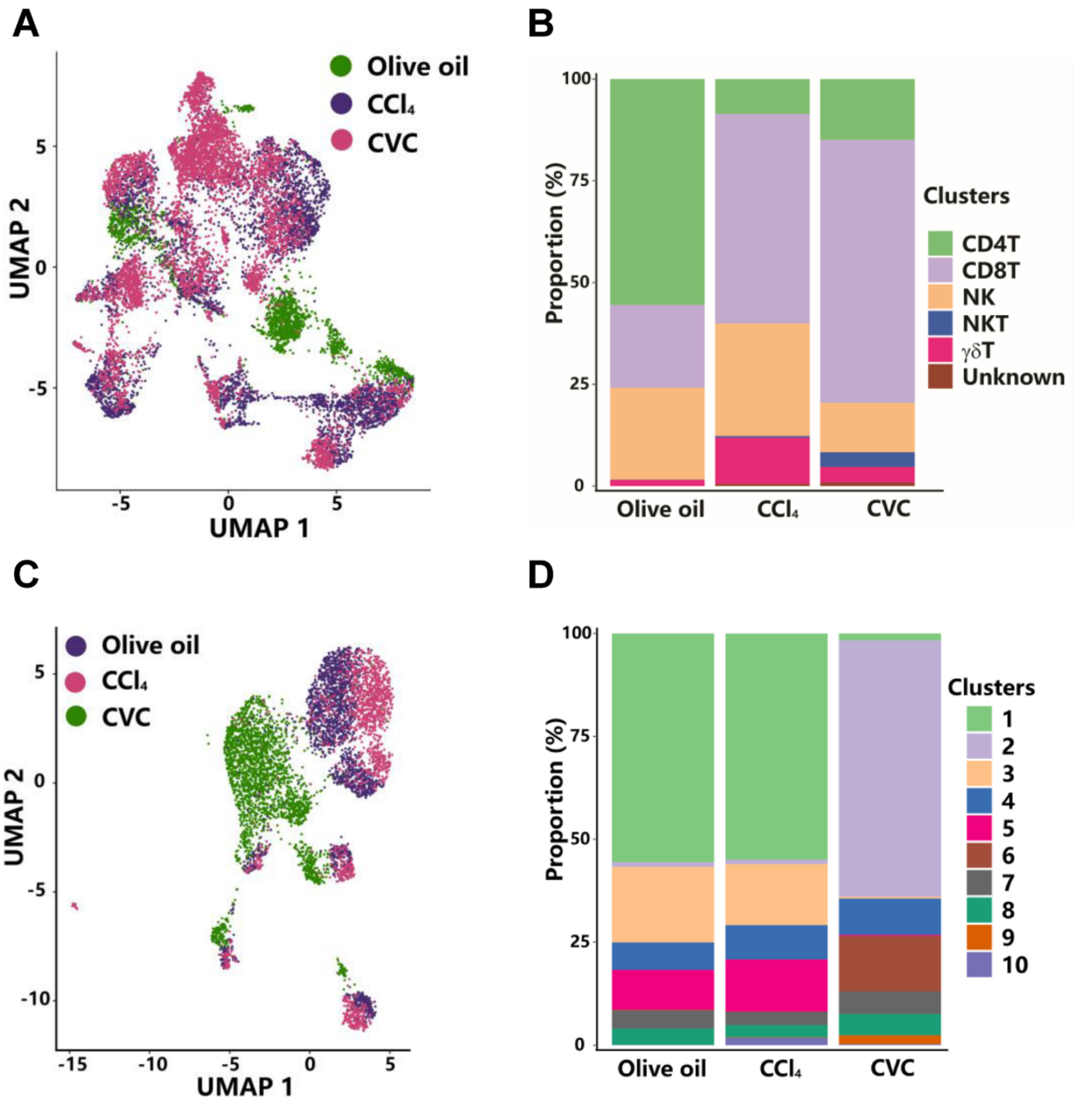

Supporting Figure S7. CVC does not disturb the T-cell and B-cell landscape in murine fibrotic livers

In the scRNA-seq data of the prevention experiment, the distribution of T cells in the three groups **(A)** and ratio changes in 6 T-cell clusters **(B)** were revealed. B-cell distribution in the three groups **(C)** and ratio changes of 10 clusters of B cells in the three groups **(D)** are shown.

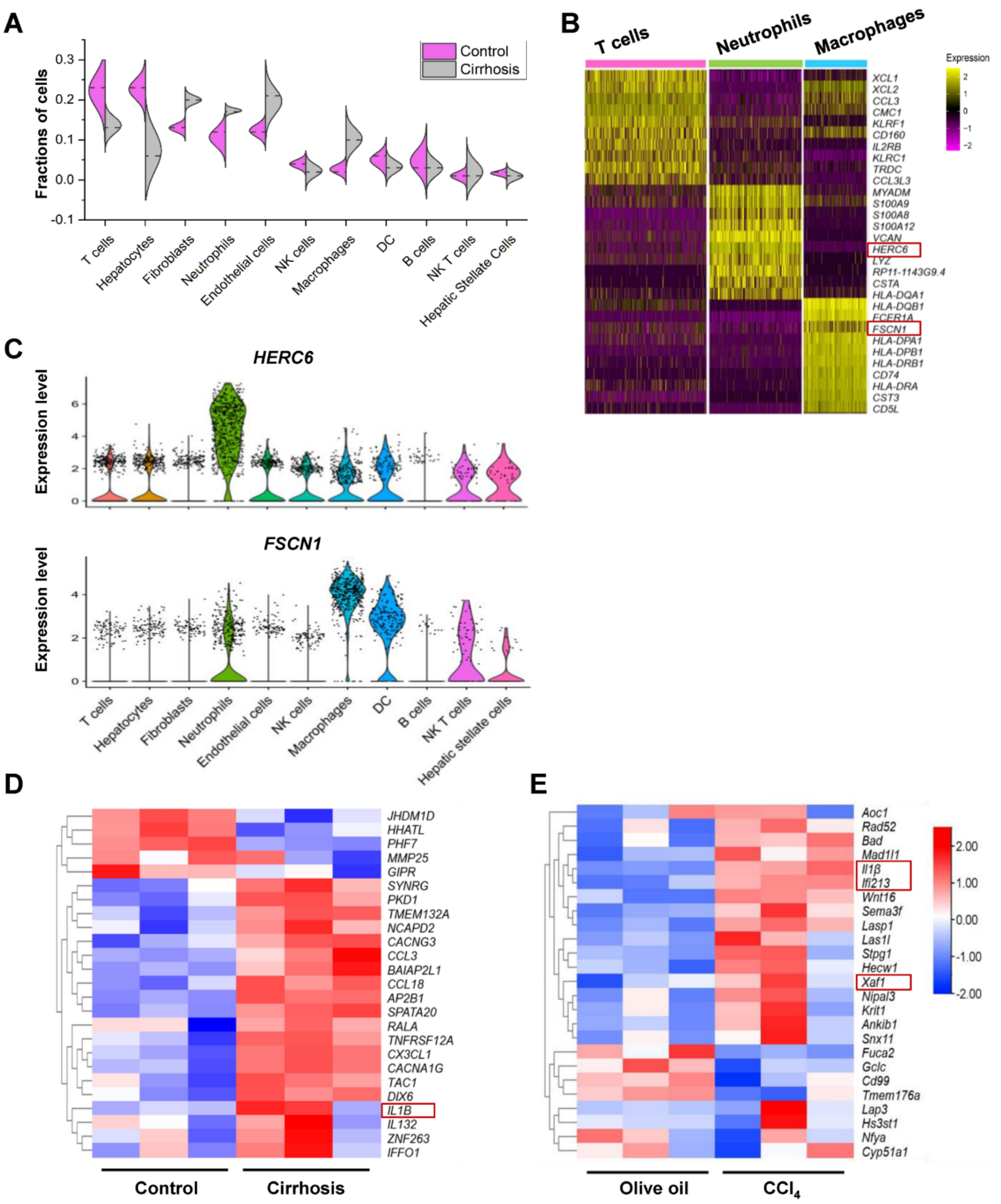

Supporting Figure S8 Bioinformatic analysis of the public GEO dataset

(A) Fractions of cell subpopulations in the livers of healthy controls and cirrhotic patients. n=5/group.

(B) Heatmap of marker genes for T cells, neutrophils, and macrophages.

(C) Scaled gene expression of *HERC6* and *FSCN1* across cell subpopulations from healthy livers and cirrhotic livers.

(D) Heatmap of differentially expressed genes in hepatic macrophages of healthy controls and cirrhotic patients.

(E) Heatmap of differentially expressed genes in hepatic macrophages of olive oil- and CCl_4-treated mice, n=3/group.

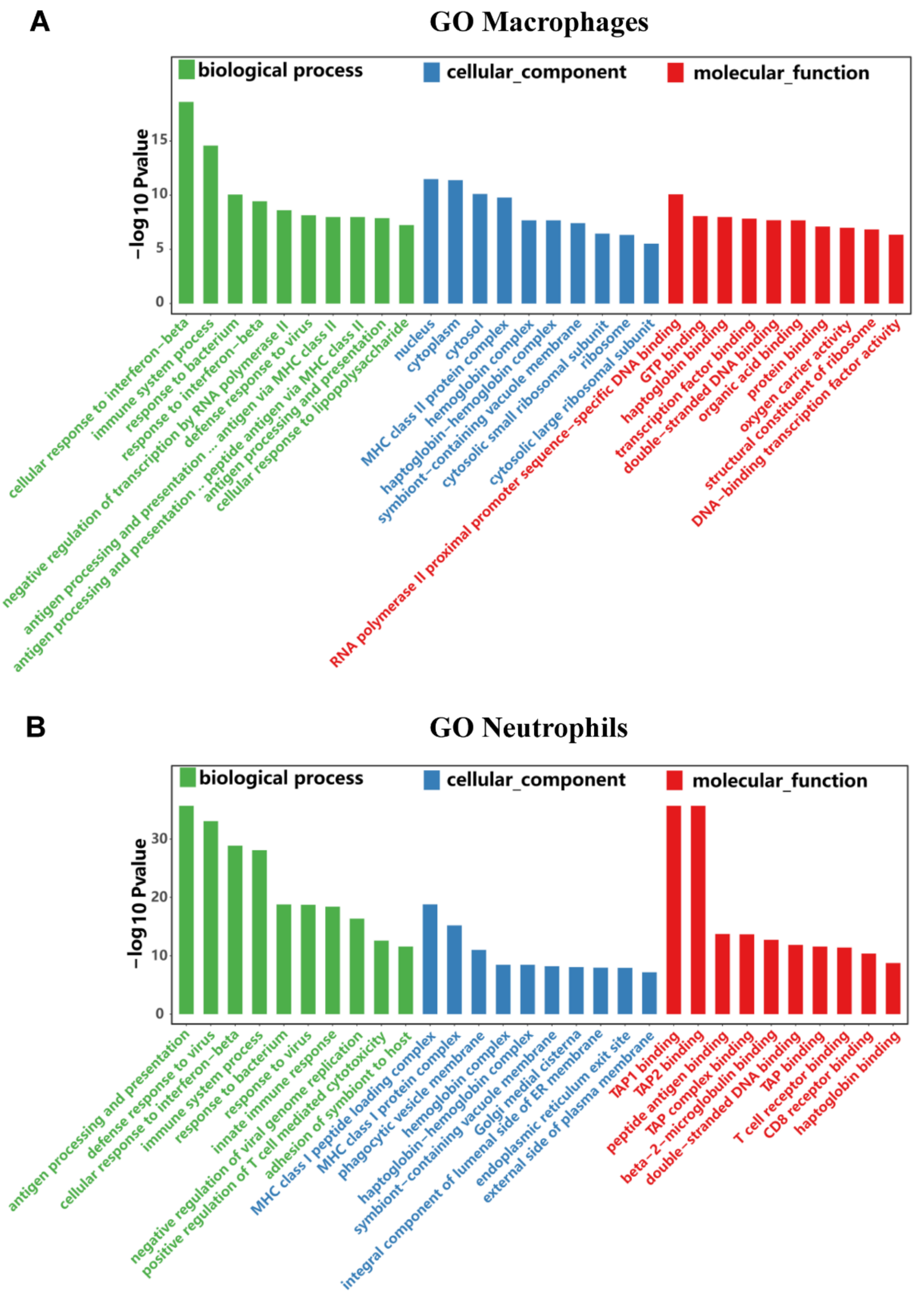

Supporting Figure S9. CVC improves macrophage and neutrophil landscapes via

potential GO signaling pathways.

(A) GO analysis showed that the biological process, cellular component, and molecular function of macrophages were restored by CVC.

(B) GO analysis showed that the biological process, cellular component, and molecular function of neutrophils were restored by CVC.

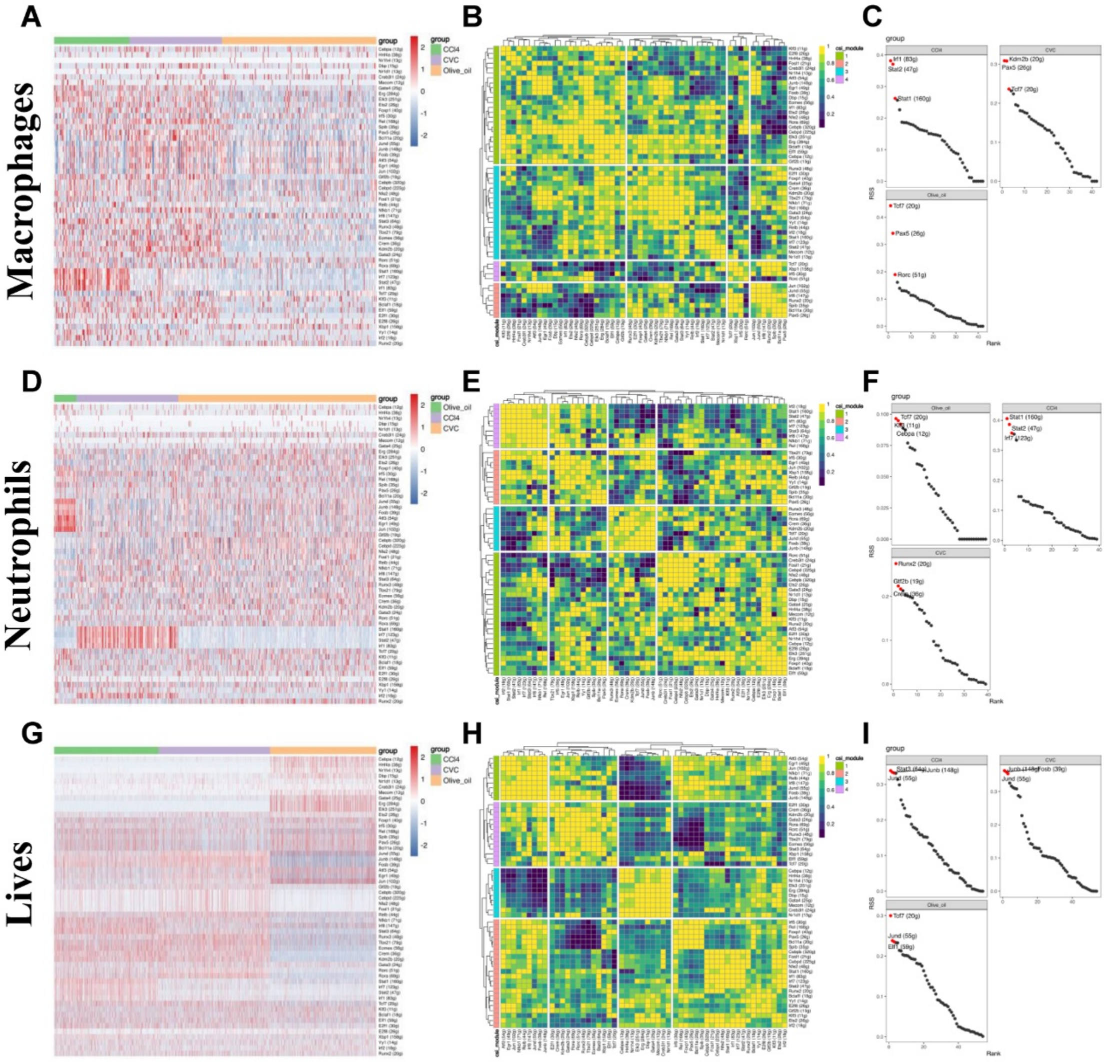

Supporting Figure S10. Regulon analysis in whole livers, macrophages, and neutrophils.

(A-C) A heatmap of RAS regulon activity in macrophages of three groups (A), the regulon CSI correlation heatmap (B), and RSS ranking plots of regulons in three groups

(C) were shown.

(D-F) A heatmap of RAS regulon activity in macrophages of three groups (D), the regulon CSI correlation heatmap (E), and RSS ranking plots of regulons in three groups (F) were displayed.

(G-I) A heatmap of RAS regulon activity in whole livers of three groups (G), the regulon CSI correlation heatmap (H), and RSS ranking plots of regulons in three groups (I) were determined.

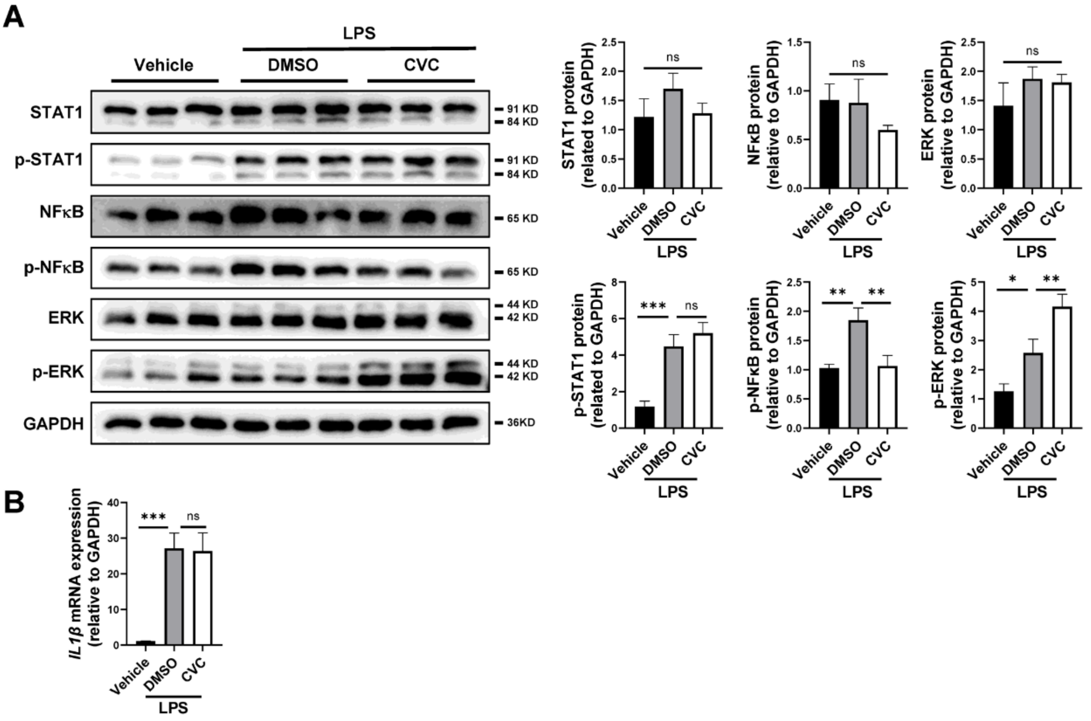

Supporting Figure S11 Insufficient CVC in regulating intracellular signaling in human macrophage THP1

(A) Human macrophage THP-1 cells were treated with DMSO or CVC for 2 hours, followed by stimulation with LPS or vehicle for an additional 3 hours. Protein levels of STAT1, p-STAT1, NFκB, p-NFκB, ERK, and p-ERK were determined by WB.

(B) The mRNA level of *IL1β* was quantified by qPCR.

*$p<0.05$, **$p<0.01$, ***$p<0.001$, ns, not significant.

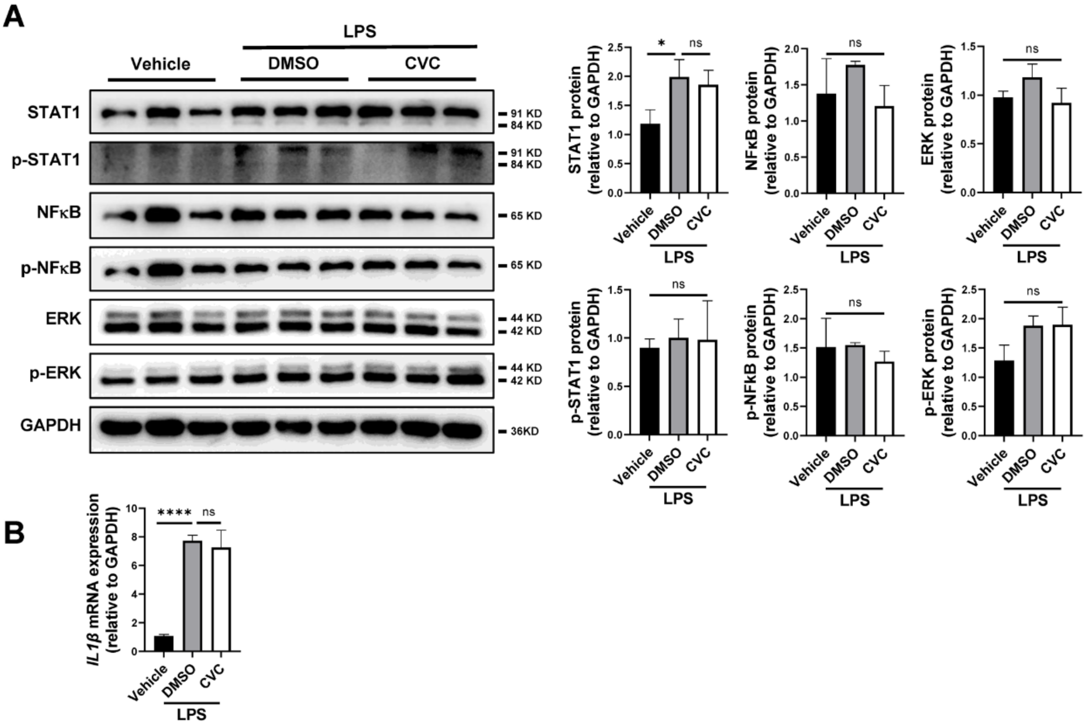

Supporting Figure S12 Insufficient CVC in regulating intracellular signaling in human HSC LX2

(A) Human HSC LX2 cells were treated with DMSO or CVC for 2 hours, followed by stimulation with LPS for an additional 6 hours. WB was applied to determine the protein levels of STAT1, p-STAT1, NFκB, p-NFκB, ERK, and p-ERK.

(B) The mRNA level of *IL1β* was quantified by qPCR.

*$p<0.05$, ****$p<0.0001$, ns, not significant.

References

1. Ramachandran P, Dobie R, Wilson-Kanamori JR, Dora EF, Henderson BEP, Luu NT, et al. Resolving the fibrotic niche of human liver cirrhosis at single-cell level. Nature. 2019; 575: 512-8.
2. Verboven E, Moya IM, Sansores-Garcia L, Xie J, Hillen H, Kowalczyk W, et al. Regeneration Defects in Yap and Taz Mutant Mouse Livers Are Caused by Bile Duct Disruption and Cholestasis. Gastroenterology. 2021; 160: 847-62.
3. Mederacke I, Dapito DH, Affò S, Uchinami H, Schwabe RF. High-yield and high-purity isolation of hepatic stellate cells from normal and fibrotic mouse livers. Nat Protoc. 2015; 10: 305-15.
